# Spirit of the Foreign Periodicals, &c.

**Published:** 1836-10-01

**Authors:** 


### Spirit of the Foreign Periodicals, &c.

On the Varieties and Pathological Differences of the Human Race.—Remarks on the Varieties of Vegetable and Animal Species.

The venerable Nestor of German medicine, Dr. Hufeland, introduces the first number of his long-established Journal for the current year, by a paper with the above title.

The attention of scientific men, says he, has of late years been much occupied with the examination of the varieties of the human race, in reference to their colour, their general structure, the formation of their skeleton, and their physiognomy. Some writers have gone so far as to speak of originally distinct families, or great tribes of mankind, and they have maintained that each large district of the earth, as it has its peculiar plants and insects, is, in a like manner, tenanted with a peculiar race of men. Man, however, is not to be viewed on the same footing as vegetables, or any of the lower animals —he is the first member of a higher grade of beings, a spirit-born existence (geist-geborner.) The more accurately that we attain to a knowlege of different tribes and nations, the more completely are we convinced of the truth, that there is only one original species of the human race, and that all men are derived from one common stock.

In reference to the question often proposed, whence have arisen the individual varieties of the human race, which we daily see in our travels? we should attend to the following considerations:—The formative effort or impulse (bildungstriebe) is subject to certain deviations or disturbances from the healthy or natural standard, in consequence of a variety of influences. Hence arise various defective directions of the primary arrangement and development of the whole, or of parts only of the being, both before as well as after birth. It has been too much the custom, hitherto, to examine the varieties of man only on the large and extended scale of nations, or of large masses of people, and to overlook the differences which may be observed among the different inhabitants of a very limited district, or members of small tribes. It would not be difficult to discover in every town and village varieties of men, as obvious and as well marked as that of the Greenlander is from the Mongolian, or either of these is from the German. We see these varieties extending and propagating themselves, and thus giving rise to other beings, in all respects like to themselves. For example, the Cretins differ in bodily formation and in mental powers from the surrounding tribes much more obviously than the negroes, or native Americans, differ from any of the inhabitants of Europe. We should also remember that the varieties of some of the lower animals, as for example of the dog race, are much greater and more conspicuous than those which we observe in the human family; and are we to believe that each of these varieties constitutes primarily and specifically different tribes, and not rather that they are all derived from one common and original stock?

In all our reasonings upon the causes of the varieties of animal tribes, we are to keep in mind that congenital defects or deviations, and even such as have been acquired after birth, may be propagated and communicated to the offspring of the individuals, and may thus become the distinctive attributes of a particular race. The physiognomy, the character, the formation, the disposition to certain diseases, we observe to pass from parents to their children, and to become distinctive marks of certain families or tribes. It is known that the descendants of a particular family, through several generations, have been characterized by having six fingers and six toes on their hands and feet; and it has been often remarked that the Jewish male children are born with shorter prepuces than the infants of other tribes.

The deviations of development which are communicable by propagation are not always manifested immediately on the birth of the individual, but often they appear for the first time at an ulterior period of growth, or in consequence of some diseased action becoming established in the system.

The two chief causes of the varieties of the human race are climate and descent. The influence of climate, under which general term we comprehend not only the temperature, the state of the atmosphere, and the prevailing winds of a district, but also its soil, its waters, its geographical elevation, its vegetation, and the means and resources of subsistence with which it is provided, is too obvious to escape the attention of any observer. Men, beasts, and plants all feel and testify its power. In the case of the human race, the hue of the skin varies with the climate. In cold regions it is fair; and as we approach the equator it becomes browner and browner, until it acquires a jet black colour.

Buchanan, in his travels through India, alludes to a tribe of black Jews, who have probably been settled in the district which they now occupy ever since the period of the captivity under Nebuchadnezzar, 3000 years ago, and who retain all the national peculiarities of their race, with the exception only of their colour, which is now quite as dark as that of the surrounding tribes.

From the consideration of the external and more obvious differences of the different tribes of mankind, Dr. Hufeland proceeds to examine the peculiarities of constitution, which not only every tribe, but also almost each individual of every tribe possesses, and the study of which is so necessary to the physician, and to the moral philosopher. He very justly remarks, that what a correct knowledge of the dispositions and inclinations is to the physician of the mind, a knowledge of the corporeal temperament and constitution is to the physician of the body.

We may classify and arrange the inward or constitutional differences of men, first in reference to their purely physical or material relations, distinguishing them into the dry, the moist, the firm, the fatty, the lean, the hot, the cold, and so forth; and secondly, in reference to their physico-psycological relations or temperaments, according to the division of Galen. Dr. Hufeland prefers to associate together these two methods, and to consider man in all his relations (in seiner totalitat), physical or corporeal, as well as moral or spiritual. It is only by following this plan that we can rightly study and appreciate the "nature of a man," or discover and describe correctly the essential differences of the human race. Dr. H. admits the following varieties of physico-mental constitutions: the strong —the weak—the fiery or hot—the excitable or sanguineous—the cold or sluggish—the compact or tough (verschlossene oder zahe), corresponding with the melancholic temperament of other authors—and, lastly, the sensitive or nervous. The last mentioned is an offspring or production of later times, and is the birthright (geburtseigenthum) of a highly educated and refined state of society. Its prominent and characteristic attributes are, the preponderance or large development of the nervous system; an unusual susceptibility to all impressions, and a consequent excitable and mobile state of the whole body; a changeableness and tendency to irregularity in all the functions, bodily as well as mental; the existence of unusual sympathies, and the disposition to sudden transitions from one extreme to another; the prevalence of phantasies and passing delusions, and the proneness to imaginary complaints, to cramps, to hypochondriasis, and hysteria. Besides the above general and comprehensive classification of constitutions, we may with advantage recognize another division, founded on the preponderance of certain functions or qualities of the corporeal frame; such as the dry and tense constitution —the lax and spongy—the lymphatic —the gastric, bilious, and atrabilious —the rheumatico-catarrhal—the psoric (characterized by a defective cutaneous secretion and growth; hence always an impure skin, and a constant tendency to eruptions on the surface)— the venous-hæmorrhoidal—the phthisical—and the apoplectic.—*Hufeland and Osann's Journal.*

The doctrines of Hufeland, in reference at least to the common origin of the various tribes or races of mankind, are those which are maintained by most of the natural historians of the present day. The influences of climate, food, occupations, &c. in modifying the external appearances, and sensual attributes of certain species, both in the vegetable and in the animal kingdoms, are very remarkable. The horticulturist can prove to us, that the crab has been transformed into the apple, the sloe into the plum; that flowers have changed their colour and have become double, and that these new characters can be perpetuated by seed; that a bitter plant with wavy sea-green leaves taken from the sea-side, where it grew like wild charlock, and transplanted into the garden, has lost its saltness, and has been metamorphosed into two distinct vegetables, as unlike each other, as each is to the parent plant—the red cabbage and the cauliflower. The different races of cabbages afford an astonishing example of deviation from a common type. It is only however by the intervention of art, and by all the niceties of the most careful cultivation that these varieties can be propagated; and we find that when the plants are neglected for a length of time, and permitted to grow quite wild, they have always a tendency to re-assume the characters of their original stock;—more especially if the soil be sterile, and not unlike to that of the sea-coast. This tendency to relapse to the original state was well exemplified in some individual plants which were seen growing in 1831 on the cornice of old London Bridge.

Even when the garden varieties of the cabbage, and of other plants can be propagated by seed, it deserves to be noticed, that these are always much less prolific than the wild individuals of the same species. Similar remarks may be applied to double flowers—the ovarium is frequently abortive, and the seeds when prolific are generally much fewer than when the flowers are single.

The following experiment narrated by M. Herbert in the Hortic. Transact. Vol. 4. illustrates forcibly the tendency of some plants to the formation of varieties.

"I raised from the natural seed of one umbel of a highly manured red cowslip a primrose, a cowslip, oxlips of the usual and other colours, a black polyanthus, a hose-in-hose cowslip, and a natural primrose, bearing its flower on a polyanthus stalk. From the seed of that very hose-in-hose cowslip, I have since raised a hose-in-hose primrose. I therefore consider all these to be only local varieties, depending upon soil and situation."

The influence of soils in causing a change in the colours of certain flowers is exemplified in the case of the hydrangea hortensis. In garden mould or compost, they are invariably red; in some kinds of bog-earth, they are blue; and the same change is always produced by a particular sort of yellow loam.

Not less remarkable are the changes and modifications which we observe to be induced in certain animals from the influence of domesticity and of other accidental circumstances. Take for example the different races of dogs. How much they differ from each other in point of size, in the length of their muzzles, in the convexity of their foreheads, in the colour and quality of their furs, and in their attributes and instincts! We look in vain in the wild state for mastiffs, harriers, spaniels, greyhounds, terriers, curs, &c.; yet all these varieties have sprung from a single race, approaching very nearly in characters to the wolf.

Different however as these varieties of dogs are in outward appearances, the one from the other, there is no foundation for the fanciful theory of Lamark respecting the growth of new organs, and the gradual obliteration of others, The greatest departure from a common type—and it constitutes the maximum of variation in the animal kingdom—is exemplified, we are informed, in those races of dogs, which have a supernumerary toe on the hind foot with the corresponding tarsal bones; a variety analogous to one presented by six-fingered families of the human race, and which has been alluded to by Hufeland in his observations. Our author has also alluded to the occasional transmission of certain new and acquired habits and qualities from parents to their offspring. The following is a very curious instance of a new hereditary instinct. "There is a race of dogs employed for hunting deer in the platform of Santa Fé in Mexico. The mode of attack, which they employ, consists in seizing the animal by the belly and overturning it by a sudden effort, taking advantage of the moment when the body of the deer rests only upon the fore legs. The weight of the animal thus thrown over is often six times that of its antagonist. The dog of pure breed *inherits* a disposition to this kind of chace, and never attacks a deer from before while running." On the other hand, there is a mongrel race of dogs employed by the inhabitants of the banks of the Magdalena in hunting the white-lipped pecari, in which the influence of a new instinct is equally conspicuous. "The address of these dogs consists in restraining their ardour and attacking no animal in particular, but keeping the whole herd in check. Now among these dogs some are found which, the very first time they are taken to the woods, are acquainted with this mode of attack; whereas a dog of another breed starts forward at once, is surrounded by the pecaris, and, whatever may be his strength, is destroyed in a moment."

The qualities and disposition of the pointer, of the retriever (which stops and brings back the game of its own accord), of the shepherd's dog, and of many others, are strictly and truly of hereditary communicability.

However marked may be the differences of size, form, and habits of different races of domesticated animals, they have all, when left to run wild, a tendency to resume a common resemblance.

"It is well known that the horse, the ox, the boar, and other domestic animals, which have been introduced into South America, and have now run wild in many parts, have entirely lost all mark of domesticity and have reverted to the original characters of their species. Dogs also have become wild in Cuba, Hayti, and in all the Caribbean Islands."

These extracts are from the second volume of Lyell's Principles of Geology; a work replete with the most original instruction, and admirably calculated to inform and delight the lover of natural history.—*Rev.*

---

### M. SERRES ON SOME OF THE LAWS OF PROGRESSIVE DEVELOPMENT IN THE HIGHER ANIMALS.

If you examine the adult skeleton, you find that certain parts of it are double, such as the ribs, the bones of the extremities, certain bones of the cranium, &c., while other parts—those namely which are situated in the line of the mesial plane—are single; for example, the spinal column, the sternum, the os hyoides, the vomer, and the ethmoid, sphenoid, and occipital bones.

This mesial *unity* appears at first sight to be opposed to the lateral *duality;* but, if we trace the progress of ossification in the young animals, we at once discover that the central or mesial parts are at first double also—the corresponding or analogous portions being strictly lateral—and therefore that the whole of the osseous system obeys one common law, the law of symmetry, or of lateral duality.

We find that in the earlier periods of life, there are two demi-crania, two demi-spines, two demi-sternums, &c. and moreover that the law of double formation affects equally the development of the softer parts. Thus there are two demi-brains, two demi-wombs, and two demi-livers. Let us examine the formation of the spine a little more attentively.

In the young embryo it is found to consist of two membranous folds, quite distinct or separated from each other, the one being situated on the right side, the other on the left side of the mesial plane. I have observed them in the young chick in about twelve hours after the commencement of incubation.—There are therefore at this early period of formation two demi-spines, just as there are two parietal, and two temporal bones. In from twelve to twenty-four hours subsequently, we perceive on the two sides of these membranous folds numerous yellowish-white points, almost quadrilateral in shape, but having their angles rounded. These are the rudiments of the vertebræ. As the osseous formation advances, the two lateral surfaces gradually approach to each other, until they meet along the mesial line, and form a uniform solid case inclosing the spinal marrow. This soldering together of the lateral halves takes place in the chick from the thirteenth to the fourteenth day of incubation. The dorsal vertebræ are united a good deal earlier than the lumbar and caudal vertebræ. Similar appearances to those now described may be seen in the young human embryo. The double line of osseous points, which are to form the central part of the sacrum, are very distinctly seen in the young embryo. Of all the vertebræ, the atlas is the most tardy in being ossified. It usually remains more or less cartilaginous until the end of the first year, and at this period we observe two lateral points or nuclei, which gradually converge towards each other, to form the body of the vertebra. If the process of development is from any cause arrested at an early period of fœtal existence, the two lateral portions of the spinal column may remain disunited, and as it were, cleft through and through, either at one or more points, or along the whole extent of its tract. If this arrested development has occurred in the bodies of the cervical vertebræ, the œsophagus may enter into the cleft vertebral canal; if, on the other hand, the sacral vertebræ are affected, the rectum will be the part engaged, and this protrusion will sometimes be so considerable as to occasion a herniary tumor outwardly. M. Lisfranc and myself have examined several cases of these anomalies. In the last-mentioned sort of malformation—namely, when the rectum protrudes between the cleft sacrum outwardly on the back, we have been enabled to remedy it partially by compressing the herniary tumor, and thus causing the discharge of all the meconium, and afterwards by maintaining the reduction of the gut by means of a compress and bandage. But a more common malformation is that, where the spinal apophyses only of the vertebræ are imperfectly developed, and remain therefore disunited: the spinal marrow and its membranes are protruded through the fissure, and the disease which has been called spina bifida is induced. In some rare cases, the whole extent of the spinal column is in this imperfect state, and the vertebral canal is open throughout from the cranium to the coccyx.

These diseases illustrate and fully confirm the law of double development of the spine; and, on the other hand, this law affords a satisfactory explanation of their occurrence.

Let it therefore be well remembered that the skeleton is formed of two analogous and similar halves; the one on the right side, the other on the left. Every person knows that the right half is somewhat more strongly developed than the left one—a phenomenon which is observable chiefly in the upper and lower extremities. This predominance is apparent even in the vertebral column; the left half of this column being rather more feeble than the right half.

This relative weakness of one side is the primary cause of incurvations of the spine, as Dr. Guerin first clearly established. In this defect of symmetry we trace the cause of the greater frequency of curvatures towards the right than towards the left side. In a hundred cases of spinal deviation, there is scarcely one, in which the spine is inclined towards the left side. The curvature to the right side does not compromise life to the same degree as that to the left side; and for this reason, that the heart and the great vessels connected with the centre of the circulation are less seriously incommoded. When the curvature is towards the left side the central organs of circulation are necessarily more impeded in their free play, and life is therefore more seriously endangered.

This is one of the many advantages of a philosophical study of comprehensive anatomy;—an anatomy which is not limited to a mere discovery of the structure of living bodies at one period only of their existence, but which carefully and minutely attends to the multifarious progressive changes which occur from the earlier stages of their development, to the full maturity of their growth. If an infant have its spine inclined towards the right side, the chances are greatly in favour of its life being saved, even although no remedial measures are used for its relief; whereas, if the deformity is on the left side, the utmost care and attention will be necessary to prevent the existing impediment to the circulation and breathing proving fatal at, or about the period of puberty.

It rarely happens that an anatomical truth does not lead to some useful discovery in pathology. The ingenious reasonings of M. Guerin have pointed out a rational and successful treatment in many cases of spinal deformity.—Formerly surgeons were in the habit of endeavouring to rectify such cases by drawing the spine, above and below the seat of the deviation, in opposite directions. M. Guerin, on the contrary, has substituted a method which acts directly on the centre of the deformity, by inducing a curvature in a direction, the reverse of that which it is intended to remedy.

We now pass to the consideration of the osteology of the cranium and chest; and in the development and formation of these parts we shall find that the same laws operate, which, we have attempted to shew, influence the growth of the vertebral column.

And first, what is the cranium? If it is, as some authors maintain, to be viewed as an expanded vertebra, or as an assemblage of vertebræ; if the encephalic cavity is nothing else than the vertebral canal dilated and "ramené sur lui-meme," if the encephalon itself is the mere continuation of the spinal marrow, then we should à priori infer, that the mode of formation and of the development of the two bony cases will be nearly the same, or at least that it will be analogous. Let us now examine whether these predictions are confirmed by anatomical enquiries. M. Serres has pursued this subject with great ability. After having, as it were, decomposed, or resolved a vertebra into its fundamental and constituent portions, he takes each of these portions and compares them successively with the corresponding elementary bones, which enter into the formation of the cranium.

In this manner he has shewn the perfect conformity in structure of the occipital bone and of an individual vertebra; and by following this analytic process, he has pointed out a similar, but certainly a much less obvious conformity, which may be traced between the anterior and the posterior divisions of the sphenoid bone, of the æthmoid bone, and even of the bones of the face, and the primary constituent portions of a vertebra. This conformity or analogy becomes less and less conspicuous, both with the cranial and with the facial bones, the farther they are distant from the cervical extremity of the spine. M. Serres admits six cranial, and two facial vertebræ. Other anatomists have differed as to the exact number of these; but the opinions of all are fundamentally the same—namely, that an unequivocal resemblance may be traced between the formation and development of the vertebræ, and of some of the bones of the head. All the lateral portions or segments of the cranial vertebræ are greatly amplified and expanded for the purpose of enveloping the brain and the organs of sense, while the bodies or central portions (for example, the basilar part of the occipital, the body of the sphenoid, the perpendicular plate of the ethmoid and the vomer) are proportionally diminished or rudimentary. Now these bodies or central portions, when examined in the young fœtus, are, all of them, found to be double or mesially divided, just as we have already shewn to be the case with the bodies of the vertebræ themselves.

The posterior part of the occipital, the two parietal, and the frontal bones, may in truth be viewed as analogous to and corresponding with the spinous processes of the vertebræ.

This analogy in the formation of the bones of the head and of the vertebræ being once established, we are provided with a key to the explanation of many of the irregularities which are occasionally met with in these bones. For example, the base of the occipital bone, and the body of the sphenoid, remain sometimes quite disunited, and a portion of the encephalon protrudes, or forms a hernia into the back of the pharynx.

If the body of the ethmoid is imperfect from an arrested development, a cerebral hernia into the nasal fossæ takes place. This imperfect state is much more frequently observed in the occipital, parietal, and frontal bones; and hence congenital herniæ of the brain are usually seen at some part of the convex vault of the cranium.

Those who wish to know further particulars on this branch of congenital pathology, will do well to consult the writings of the St. Hilaires, father and son.

The primary duality of the spine, of the cranium, and of the face, explains the etiology of a multitude of congenital diseases. In truth, all the organs or parts of the body, which in the adult subject are found to be single, and which are situated in the mesial line, were originally double, or cleft in two in the middle, and became united, or, as it were, soldered together, at some definite period of intra-uterine life. If this law holds good in organogeny, it must be equally so in pathology, and it is manifest that the causes which, in the cases alluded to above, operate on the spine and on the cranium, may act in the same manner and degree on the chest, abdomen, and on the organs of generation.

If we examine the sternum at different stages of fœtal life, we find a beautiful illustration of this law of mesial duality. In the very young fœtus, the sternum is quite cartilaginous, and then it is laterally double, or cleft along its mesial line. Gradually we observe a double row of osseous points or nuclei, which in course of time coalesce, and thus the mesial line of separation is obliterated.

Sometimes we find that there is a large cleft or aperture in the centre of the ossified sternum. This imperfection is attributable to the progressive development of the bone having been partially, or only at one point arrested. When this cleft is considerable, the heart may be protruded through it, and thus the disease, which has been designated ectopia cordis, is induced.

In pathology, as in other sciences, well-established facts always tend to explain and illustrate each other. A fact which, considered by itself, may appear to be very curious and strange, becomes, if associated and viewed along with other analogous facts, simple and of easy explanation. This is the case with the congenital disease or imperfection, the fissure of the palate—a disease for the remedying of which, the operation of staphyloraphy has been contrived.

Now what is the true nature or cause of this fissure? It is in truth the result of a progressive and normal development of the bone having been stopped or arrested at some period of fœtal life. The same holds true of hare-lip, of fissure of the alveolar process of the maxillary bones, of spina bifida, and numerous other congenital malformations. The organic lesion, in all these instances, proceeds from one and the same cause—arrested development.

In one case of fissure of the sternum, which fell under M. Serres' notice, there was also a mesial fissure or division of the diaphragm; the aorta and œsophagus were displaced by a hernia of the intestines which had taken place through the unnatural opening. Olasner has recorded a case, somewhat similar to this one, in which the child lived to nine years of age.

In the early stages of embryotic existence, the abdomen, throughout its whole length, is quite open, and the greater part of the bowels are lodged within the sheath of the umbilical cord. It not unfrequently happens that this open state of the abdomen does not close entirely by the period of birth; and hence the origin of most cases of congenital hernia. When the aperture is large and the protrusion is considerable, the lesion is rarely curable.

Such cases are very common, and numerous examples of malformations of the abdominal parietes have been recorded by all the systematic writers on morbid anatomy. The rationale of these irregularities of formation is the same as we have already alluded to in reference to the malformations of the osseous system. The lateral halves of the abdominal parietes are at first quite apart and separated from each other; and if, therefore, the progressive and ulterior development is arrested, the child at birth will be found to exhibit an imperfectly closed abdomen. The two halves of the central or mesial organs of the body gradually approach each other, until they coalesce, and form one continuous and uninterrupted structure. Such are a few illustrative examples of that law of symmetry, which Meckel has denominated the "lex Serriana" (in compliment, we presume, to M. Serres), and which so satisfactorily explains many of the congenital deviations of structure. We now proceed to investigate another topic of organogeny, which has hitherto perplexed most philosophic anatomists.

On looking at the osseous and the other organic systems of the body, we find that many of them are hollowed out into cavities, perforated with apertures for bloodvessels and nerves, and traversed with canals, which either receive and convey the fluids necessary to life, or transmit these into the surrounding peripheral parts.

Now what is the rational explanation of these formations? Let us take, for example, the vertebral column, and examine the various apertures which it exhibits for the passage of numerous bloodvessels and nerves. On the side of each vertebra, there is a semi-elliptic hollow or excavation, which, being applied to a similar hollow on the body of the next vertebra, forms an aperture for the passage of a spinal nerve and some bloodvessels. The formation of these vertebral apertures is effected in the same manner as that of all other apertures or perforations of the osseous system—namely, by the re-union of different portions of the bone during its process of development. For example, the foramen magnum of the occipital bone is formed by the conjunction of the four pieces, of which the bone originally consisted; the superior and inferior maxillary holes of the sphenoid bone result from the coalescence of the two halves of the alæ, and the same is true of the foramina rotunda and ovalia, and also of the meatus auditorii. Such is the mode of formation of all the apertures and passages which we observe in a mature bone; the bone originally consisted of several pieces, and as these pieces approached each other in the process of development, a cleft or vacant space was left.

The same law holds good in reference to the perforations which we find in many of the soft structures and organs of the body. The septum of the auricles is, every one knows, cleft in the fœtus. Now this septum is, like the diaphragm, formed of two pieces developed apart from each other; the one portion descending from the upper wall of the auricle (which is originally a single sac), and the other portion ascending from its lower wall; and, as these approach each other, a cleft or unclosed space, the foramen ovale, is left. This cleft or aperture is always proportionately larger in the young than in the advanced fœtus.

In the same manner, we may explain the formation of the various apertures through the diaphragm for the passage of the vena cava, the aorta, and the œsophagus; and not only of these apertures, but also of the pupils of the eyes, and of the openings of the mouth, the anus, the vagina, &c. The margins of all these openings were at one period of embryotic life imperfect; and all these openings, without exception, were formed by the "adossement" or meeting together of two elliptic or semicircular muscles. Some of these parts or structures, which are normally open and perforated in the mature fœtus, were primarily formed of serous-looking membranes, which were quite closed through all their extent. If the portion which corresponds to the opening remains longer than the period assigned to it in the series of developments, the child is born with one or more of its passages imperforate.—(These statements are, it must be confessed, far from being easily intelligible.—Rev.) This malformation is not unfrequent at the anus and vagina.

What has been stated in reference to the formation of the apertures in the bones and other parts of the body, may be applied to the explanation of the development of the various canals, as of the nasal, intestinal, urinary, &c.; and, in short, what is a canal, but merely a lengthened or prolonged aperture? The walls of the canal were at some period of fœtal life composed of two or more detached lateral portions, and these portions, gradually approaching each other, at length coalesced, so as to form a continuous and uninterrupted passage.

Thus the nasal canal has been formed by the meeting together and union of the superior maxillary, the vomer, and the turbinated bones. The central canal of the spinal marrow has arisen by the union, anteriorly and posteriorly, of the two lateral halves of the medulla, leaving a space between; and the intestinal canal is developed in a similar manner—by the concentric coalescence of separate lateral portions—as has been admirably described by Wolf.

The mode of formation of the urethra, also, is quite the same; and for several reasons I select this canal, to offer a few general observations on this curious subject of organogeny.

When the pelvis is still unclosed in the young embryo, the canal of the urethra is cleft throughout its whole length —the two lateral halves of the penis and of the clitoris are separated from each other, and the perineum is open along its mesial line, where the raphe is afterwards situated. These two halves of the genito-urinary organs gradually approach each other, and at length meet and coalesce about the time when the two ossa pubis become united. This coalescence takes place on the upper surface first, and afterwards on the lower. Anterior to this event, there is in fact no distinction of sexes; and all young embryos are alike.

When the union has taken place, the two branches of the clitoris and of the penis present so obvious a projection, that at this period—from the fortieth to the fiftieth day—all fœtuses appear to be males. But afterwards, as the fissure of the perineum contracts, and as the two halves of the urethra are approaching each other, a person might suppose that every fœtus was female. This second deception is manifested about the end of the second month of embryotic life.

We thus discover how it is that, at first, there is no recognizable distinction of sex; and that subsequently all fœtuses appear to be male, and then to be female, before the complete development of the organs takes place. If, therefore, the development of these parts is arrested at one or other of these periods, it is not surprising that a female child may at birth exhibit the appearance of the male generative organs, and, on the other, that a male child may exhibit the appearance of the female organs. This is the key to the explanation of the different sorts of hermaphrodism. The malformation of hypospadias (in which the canal of the urethra is more or less imperfectly closed) is readily explicable by what has been already stated. All male fœtuses, at one period of intra-uterine life, are affected with hypospadias, and, therefore, if the state of parts which constitutes this imperfection remains at birth, the child is born with a urethra more or less open along its lower surface. The open state or fissure of the canal may occur either near to the glans, or in the middle, or quite at the root of the passage in the perineum; and hence the varieties of the malformation which have been described by authors.—*Gazette des Hôpitaux.*

---

### The Siamese Twins.

They are at present 25 years of age, of small stature (being about five feet in height), and inactive constitution. Their physiognomy is strictly Chinese; their complexion is dark and glossy; their hair is long, black, and in tresses. The right twin is stronger than the left one, who seems, therefore, to be somewhat dependent on, and led by the former. Their heads, proportionate in size to the rest of their bodies, are sufficiently well formed; their foreheads are straight and moderately elevated. The bond of union between these twins consists in a tegumentary commissure, situated at the base of the sternum, and extending from thence downwards for two inches and a half, or three inches in length.

This commissure is susceptible of some elongation, and it may even be slightly twisted upon itself, without causing any pain or uneasiness to either twin. It is about two inches thick, and at its lower part is seen a single cicatrix, which is the umbilicus. While they were young, they could move and turn round upon each other; but since their tenth year, their movements have been much more limited. Even now they have a certain degree of liberty, as they can both readily lie on their sides at the same time, and take the position which they find most easy. The left twin has rather less freedom of movement in his limbs than the other; his right shoulder projects considerably, and the arm of this side is always placed behind his brother's body.

The twins have a marked aptitude for most bodily exercises; they are fond of the chase, and in America they have hunted the deer, and have even been known to have killed the game with their own hands. Both of them can use fire-arms. The right twin can fire a fowling-piece with either of his hands; the left can pull the trigger with his left hand only. It is truly wonderful how admirably they co-operate with each other in the various manœuvres and exercises which they perform. They can swim as well as almost any single person. Their sleep is simultaneous; and one has never been observed to dose, without the other immediately doing the same. They almost always awake at the same time; but, whether this proceeds from the movements of the one first awake disturbing the other, is not exactly known. They are very fond of smoking.

With respect to the state of their alvine and urinary excretions, we are informed that these functions do not always take place simultaneously; the French narrator adds—"mais ils ont desiré que l'on n'insistat sur ces questions en presence de dames (!) qui assistaient à l'examen. Ce sentiment de pudeur ne se dement pas chez eux, et est tres prononcé."

When one of the twins was bled, they both fainted together. It has been stated that, some time ago, there was a quarrel between them, one desiring to bathe, and the other objecting; but this statement is quite erroneous, as there has been uniformly the most perfect concord and unanimity existing between them on all occasions. It is however quite true, that sometimes for several days together they do not speak a word to each other. Their intellects are considerably developed; they speak the English language with facility, and the French also moderately well. They are fond of painting, music, and poetry. Byron's works are great favourites with them. Sometimes they sing together. The memories of the two seem to be equally good; they will play at chess and at draughts with strangers, but never with each other.

When carried over to America, the captain of the ship attempted to benefit himself by exhibiting them to the public; but they, knowing that they were in a free country, claimed the protection of the laws, and ever afterwards they have received themselves the profits of their exhibition.

The exact nature of the cord or bond which unites the Siamese twins, has not been satisfactorily determined. It is uncertain whether it is a mere tegumentary ligament, or whether it contains within any communicating canal between the abdominal cavities. This question has been canvassed in England and in America by Drs. Anderson, Mitchell, and Bolton, who all agree in believing that there is a herniary sac or canal. The two former are of opinion that various viscera, as the stomach, spleen, arch of the colon, and a portion of the liver, may be protruded into the canal; while Dr. Bolton thinks that the arch of the colon only is ever so displaced. It appears however to us to be very doubtful whether there is ever a genuine herniary protrusion of any of the abdominal viscera. The absence of all uneasy feelings in the various draggings to which the commissure or connecting band is subjected, after as well as before meals, leads us to believe that only the suspensory ligaments of the liver in each, imbedded in a quantity of cellulo-fibrous tissue, and both terminating at the common umbilicus, is contained within the commissure.

It has been asked, whether either of the Siamese twins has ever expressed or indicated by their behaviour the existence of any sexual propensities.—There appears to be no exact information on this point; but the French writer states that he has observed them to exhibit "une sorte d'emotion" in the presence of females, and that they invariably displayed "des prevenances marquées" to the fair sex. He reserves however the satisfactory solution of this curious problem to "the romantic sagacity of some of those Amazons who run after every thing that is new and marvellous!"

In conclusion, the narrator alludes to the possibility of disuniting the two brothers by a surgical operation; but it is almost needless to discuss this subject, as it seems that they have formally expressed their resolution "de demeurer toujours unis."—*La Lançette Française, et Gazette des Hôpitaux.*

The names of the twins are, we find, Eng and Chang. They had an attack of ague in America. The paroxysms and intermissions were simultaneous in both. The minute particulars of symptoms, as to the beats of the pulse, the number of respirations in a minute and so forth are not detailed. Most fortunately the twins seem from all accounts to have an exact similitude of tastes, feelings, and desires.

---

### Case of Twins; the second child born four days after the first.

A young woman gave birth on the 1st of April to a female child, which was weakly, but seemed to have attained its mature growth. The placenta was expelled soon afterwards, and the patient was quite comfortable, although every now and then she felt motions, as of another child in utero. (It is not stated that the medical attendant examined the abdomen with his hand after the delivery, as he ought on all occasions to do, to ascertain the state of the uterus.) On the fourth of April, this woman was again seized with sharp labour pains, which, after lasting for several hours, expelled a vigorous healthy child. The after-birth followed in the course of a short time.

It is not certainly easy to account for the unusual delay which occurred in the present case. It deserves to be noticed, that previous to the first delivery a hæmorrhage had occurred.—Now it seems probable that one only of the placentæ had become partially detached at the time, and that this partial separation brought on the expulsion of one fœtus a short time before the full period of gestation had been completed. That the second child might remain in utero longer, was not improbable, seeing that it had an independent placenta, which seems to have been quite unconnected with the other one. The learned reporter alludes to the possibility of the children being the products of two different conceptions; but influenced by the statement of the woman herself that she had indulged in connexion only once, he rejects the idea of superfœtation. It appears, however, that the father was very unwilling to claim the paternity of both children, and endeavoured to escape the expence of maintaining two, on the ground that he had cohabited but once with the the woman.—*Wildberg's Iahrbuch der Staatsarzneikunde.*

---

### REMARKS ON OSTEO-MALACIA, WITH CASES.

Cath. Ell, of a naturally vigorous and robust constitution, had enjoyed good health until she was upwards of sixty years of years. During the greater part of her life she had lived as an active servant in a family at Heidelberg, and at the age of fifty she had been admitted an inmate of one of the hospices there. The earliest symptom of the disease was the occurrence of pain in the loins: the pain became more and more severe, and it gradually extended over the whole extent of the back and also to the extremities, so that the walking was at first very difficult, and at the end of four months quite impracticable. The patient therefore was obliged to remain constantly in the horizontal position. By degrees the different joints became permanently flexed, so that both the upper and the lower limbs were kept continually bent upon and applied to the body, and could not be withdrawn. The fingers were contracted quite into the palms of the hands, and the patient was thus rendered utterly helpless.—About the sixth or seventh month after the commencement of the disease, a curvature of the spine was first noticed. About this period the pains became more severe, and were so much aggravated on the slightest movement, that upon making any exertion the poor woman could not refrain from screaming out. The appetite however remained quite vigorous, and indeed it did not at all abate until the last two or three months of her life, during which time she took very little food, but was distressed with an intolerable thirst. The excretions, with the exception of the urine, which was voided more frequently than in a state of health, were perfectly normal. The progress of the case is not detailed, and we are only informed that after two years of deplorable suffering the poor invalid died. The limbs, which had during the whole of this time been kept constantly bent and contracted upon the body, were relaxed after death, and could be brought into their natural condition. The body emaciated to the last degree, had become shortened nearly a foot and a half.

The great visceral organs, all more or less pale and reduced in bulk, did not exhibit any marked traces of disease. The mesenteric glands, the thoracic duct, and the lymphatic vessels, were carefully examined; but no lesion was detected in any part. In short, the soft parts of the body may be said to have been normal, at least in point of structure.

The cranial and facial bones did not present any morbid change. The thoracic and the upper part of the lumbar portions of the vertebral column formed a conspicuous curvature to the left side, and lower down the spine was curved in the opposite direction. The texture of the vertebræ, and more especially of the lumbar ones, was unusually soft and yielding: their cells were very large and were filled with a reddish fluid. The intervertebral cartilages were in a healthy condition.

The thorax was inclined quite over to the right side; on the ribs there were more than twenty abnormal articulations, (sur les cotes il y avait plus d'une vingtaine d'articulations contre-nature). Of these articulations those on the right ribs were nearer to their vertebral extremities than those on the left ones: they were formed by a fibrous mass of thickened periosteum, and were so flexible that they could be readily moved in all directions. Several of these articulations were very near each other, being not further apart than an inch or so. The sternal and spinal ends of the ribs did not exhibit any appearance of enlargement, as is usually seen in cases of rachitis. So yielding was the texture of the ribs in consequence of the extreme tenuity and fragility of their outer lamellæ or compact shells, compared with their cellular or spongy tissue, that by moderate pressure with the fingers, they could be easily broken or crushed into different pieces. Of all the bones, the sternum presented the most striking "ramollissement:" it was rather thicker than usual, but it might be crushed between the fingers or cut with a knife, with the greatest ease, the outer shell or lamina being very thin and fragile. The bones of the pelvis were so soft, that they could be readily bent, and when firm pressure was made on the right and left ilium at the same time, the two opposite sides of the pelvis were approximated to a certain degree.

The crista of the os ilium was exceedingly soft, and its outer lamina was very thin and perforated in several places. Notwithstanding these great changes in its osseous walls, the dimensions of the pelvis were not, we are told, perceptibly altered. The bones of the upper and of the lower extremities had almost quite escaped the effects of the disease; for with the exception of the extreme "gracilité" of their texture, they were scarcely, if at all affected. The long bones were slightly bent, but so slightly that it could not be said that they were deformed from disease; and their condyles were not at all enlarged, as is usually the case in rickets. The scapulæ exhibited a morbid character, intermediate between the softening of the pelvic bones, and the tenuity of the long bones; their bodies being exceedingly attenuated, and their edges and processes being considerably softened.

In concluding the report of this case, it may be remarked that all the bones of the trunk were more or less tumefied, and that this change was owing to the increase of their spongy or cellular texture at the expense of their compact walls. The vertebræ had undergone this morbid lesion in the greatest degree, and as they had to bear the superincumbent weight of the head and body, they gradually gave way to one side, and thus was occasioned the double curvature, which we have mentioned.

*Case* 2. Cath. S., 36 years of age, born of healthy parents, had enjoyed very good health until about her 30th year, at which time she was pregnant with her first child. During the greater part of her pregnancy she suffered from most severe pains in the sacro-lumbar region. These ceased after delivery, and her health seemed then to be re-established. Two years subsequently she was again pregnant, and the pains returned with even more severity than on the former occasion, and they became so aggravated that she could not walk, but with the greatest difficulty. After delivery, they abated for about two months, when they were renewed, and then extended to the chest and to the upper and lower extremities. About this period it was remarked by this patient's acquaintances, that her height was very sensibly diminished. She became pregnant a third time, the pains continuing until the latter half of this pregnancy, after which they ceased almost entirely. The child was well grown and healthy. The health of the mother remained good for nearly two years until the Winter of 1832, the only inconvenience of which she complained being a sharp pain in the region of the sacrum whenever she stooped or took active exertion. She was admitted into the Heidelberg Hospital in Feb. 1833, at which period the pain in the back was very severe, and had extended upwards along the spine, and also over the greater extent of the pelvis. Her breathing was considerably distressed, and in consequence of the excessive tenderness of her feet, she could not walk but with difficulty. On examining the spine, it was found to be considerably distorted, (the nature of the distortion is not stated,) and the sacrum was bent inwards, and was very tender on pressure. Her general health gradually decayed, and she died in a state of extreme marasmus.

The examination of the body appears to have been most imperfectly performed; at least no detailed report is given by the author.

The chemical analysis of the ribs and vertebræ shewed that "the proportion of their organic and inorganic constituents was sensibly different from their condition in health. The bile and the blood contained a large quantity of phosphate of lime, and of other phosphatic salts."

### *General Remarks on the Osteo-malacia of Adults.*

As yet we have not sufficient data to enable us to judge of the influence of climate, or other causes, on the development of the disease. It has been remarked by many observers, that it is not more frequent in those places where rachitis is endemic. Sedentary employments, especially if combined with imperfect nourishment, certainly predispose to that cachectic state of the system, which is apt to induce softening of the bones in adults. Women, and especially those who have been recently child-bearing, are much more subject to it than men. Instances are, however, on record of its occurring in young females, and also (but this is still more rare) in women of very advanced years.

The male sex is not exempt from the disease, as appears from the writings of Meckel (Manuel d'Anatom. Patholog.), Saillant (Ancien Journal de Med.), and others. The greater number of the cases among married women have occurred between 30 and 40 years of age, and in persons who have been exposed to destitution and distress. It is very rarely observed among the respectable classes of society. The same remark is applicable to the cases which have been seen among male patients.

The characteristic symptoms of the disease are, sharp pains in the lower part of the spine and through the pelvis, difficulty in walking, great debility and emaciation, deformity of some part of the osseous system, and, lastly, an abnormal condition of the urine. The pain is at first felt at one part only of the spine, usually over the lower lumbar vertebræ and the sacrum, and it is always aggravated by any motion or exercise. By degrees it becomes more and more continuing and severe, and it extends from the originally suffering region upwards along the spine, and also to the upper and lower extremities. Occasionally, there are intermissions of the disease for several months at a time; but, in almost all cases, when once the cachectic state is induced, the alteration in the composition of the bones returns at some future period, and becomes more and more complete, until the poor patient is hopelessly bedridden. In this miserable condition the patient may live for many years, unless, as is often the case, the breathing become seriously affected, and hydrothorax, or some other fatal malady is established.

The organs most apt to become diseased are certainly those of circulation and respiration, and to the distress thus induced is generally superadded a slow consuming fever, which is speedily followed by general atrophy. Previous to the occurrence of this febrile state, all the great functions of the body, and especially those which appertain to the generative organs, are usually performed as in health; the catamenia are regular, and the woman is as apt to conceive as if no morbid process was going on. The pains of osteo-malacia are always much aggravated by any motion of the body, and often they are accompanied with cramps of the limbs, and with a most distressing sense of anxiety and constriction of the chest, depriving the patient of rest during night or day. We have said that, in a large majority of cases, they are first felt over the sacrum and shooting through the pelvis; more rarely they commence in one of the lower extremities, and thence extend to the spine. They have this peculiar character, that the gentlest touch or slightest pressure on the bones, more especially on those of the pelvis, induces and aggravates them to a degree quite insufferable.

The patient becomes weaker and weaker, and at length loses all wish, even although the power remained, of moving from his bed. Often a long period elapses before the true nature of the disease is even suspected, and in deed in most cases, this knowledge cannot well be obtained, until the deformity of some of the bones has actually taken place. The spine first, and then the pelvis, are most early and most seriously affected. The dimensions of the pelvis become gradually more contracted, in consequence of the sinking in of the sacrum and of the ossa pubis, and also of the ossa ilii towards each other, until at length the delivery of a full-grown child, even by means of the perforator and crotchet, is rendered quite impracticable. Such, indeed, has been the history of most cases, where the Cæsarean operation has been found necessary. The fact of a woman retaining the ability to conceive, indicates how little the general health of the system sympathises, even when the morbid changes of the bones are most extensive and most serious.

With respect to the urine in osteo-malacia, it deserves notice that, in the very early stages of the disease, this secretion usually exhibits some marks of abnormal change; it becomes more or less decidedly turbid, and it deposits a whitish sediment, which is found to consist chiefly of phosphate of lime. This character of the urine generally continues during the whole progress of the disease.

In addition to the symptoms of osteo-malacia already enumerated, we may allude to others of occasional occurrence, such, for example, as permanent contraction of the joints, unaccompanied, however, with any enlargement of these, such as we observe in gout and chronic rheumatism. Sometimes the bones of the extremities are unusually friable, so that they break on the slightest exertion, or even by the force of mere muscular contraction. Cases are on record, where several such fractures have taken place in the course of the disease.

The functions of digestion and assimilation are often but little deranged, even when the patient has been bed-ridden for a series of years; not so with the functions of the thoracic organs, as already noticed. The breathing is almost always more or less distressed—at first, perhaps, from mere sympathy, or perhaps from irritation of the spinal marrow, and ultimately from positively organic changes in the structure of the lungs.

Mr. Wood, in the Med. and Phys. Journal for Oct. 1801, has pointed out the influence which the function of lactation appears to exert on the progress of osteo-malacia. He remarked that the disease in no case became developed at the commencement of suckling, and that, if it was already formed, its course was usually arrested during its continuance.

The anatomical characters of the bones, which have suffered from osteo-malacia, are these: not only the apophyses, but also the bodies or shafts of the bones, are more or less swollen and increased in volume. The "osseous mass" is, nevertheless, diminished; for the compact substance, or what is called the shells of the bones, are greatly attenuated, and the degree of this attenuation is proportionate to the duration of the disease. In the long bones, the compact parietes or shells sometimes do not exceed half a line in thickness. The spongy or cancellated structure also undergoes certain changes; the cells become much larger than in health, and the medullary canal is wider and altogether more dilated; in consequence, no doubt, of the absorption which is constantly going forward.

The osseous texture, therefore, is entirely changed, and this change is evidently not the result of a mere deficiency of the earthy constituents of the bones, although perhaps, at the commencement of the disease, this deficiency constitutes its most characteristic feature. The colour of the affected bones is always much darker than in health; sometimes it is almost black. They usually feel greasy to the finger; and, when examined, they are found to contain a large quantity of bloody fluid, and of a thin and reddish adipose matter. When boiled in water, they readily become still softer. The consistence of the bones affected with osteo-malacia necessarily varies in different cases; some may be bent, others may be cut with the knife. Although, in most cases, they are much more easily broken than healthy bones, this fragility is attributable rather to the extreme attenuation of their compact shells or outer lamellæ, than to any excess in the proportion of their inorganic to their organic component parts, or to any *softening* (as the term osteo-malacia implies) of the general osseous texture; "ils se rompent plutôt qu'ils ne se cassent." The curvatures and distortions in genuine osteo-malacia are much more frequent, and more complete in the spine and pelvis, than in the long bones of the extremities. The sacrum becomes at first inclined, and ultimately quite convex forwards, the rami of the ossa pubis fall together, so that the symphysis forms a sharp projection, and the ossa ilii also become approximated towards each other. The inlet of the pelvis is thus most seriously diminished in all its dimensions, and the head of the fœtus may be irreparably detained beyond even the assistance of the perforator and crotchet. The bones of the cranium and face are of all bones the least subject to the morbid changes of osteo-malacia: they were however affected in some degree in the famous case of the woman Supiot. Hitherto the examination of the fractures and false articulations occasionally found in patients who have died from osteo-malacia has not been sufficiently complete, to enable us to give any very satisfactory details. The periosteum is generally more or less thickened, softer than in health, and also less firmly adherent to the affected bones.

In conclusion, the author remarks that in every case of osteo-malacia the periosteum and the medullary substance have been found more or less affected with change; that this change has been observed in those cases where the bones had not yet become diseased, but in which the symptoms only of the malady had been manifested; and that the earliest symptom is pain in the part which primarily suffers. Reasoning upon these data, he is induced to believe that the disease commences in, and is perhaps dependent upon an affection of the investing membranes of the bones. At first the inorganic elements of the bones are deficient, and subsequently the regeneration of the organic elements is involved in the morbid changes. As the disease advances, there is usually interposed in the osseous tissue an excessive quantity of discoloured adipose matter. It has been conjectured by some pathologists that osteo-malacia depends upon the presence of acrid fluids in the blood; but an argument against this theory may be derived from the consideration that the disease does not affect every part of the osseous system equally; the spine and the pelvis are the parts which most frequently and most seriously suffer. The doctrine of the local origin of the disease, (induced, we doubt not, by some unknown cachexy of the system,) already alluded to, accounts more satisfactorily for some of its features, such as its attacking persons apparently in good health, the occasional intermissions in its progress, &c. &c.—*Commentatio de Osteo-malacia. H. Proesch. Heidelberg*, 1835.

---

### RADICAL CURE OF HERNIA.

Professor Gerdy has proposed the following plan. Having reduced the hernia, he pushes a fold of the integuments up the canal, so as to form a short cul-de-sac, like the finger of a glove, which plugs and obstructs the passage outwardly. He then fixes the integuments in this position by several sutures, which are to attach the bottom of the cul-de-sac to the parietes of the herniary canal or tube, and which by inducing inflammation, are expected to cause the two surfaces to adhere permanently together.

Lastly, he excites inflammation on the outer surface of the invaginated fold of integuments by dabbing it with the liquor ammoniæ, in the hope of inducing an adhesion of its parietes to each other.

Mr. Gerdy has practised his plan on two patients, one of whom has been entirely cured of his hernia.

M. Larrey however, to whom the Academy referred, reports unfavorably of M. Gerdy's proposal.—*Archives Generales.*

---

### THE SMALL-POX, AS OBSERVED IN THE HOSPITAL AT VIENNA DURING THE YEAR 1835.

The number of patients affected with small-pox in the hospital during Jan. was 52, during February 64, during March 68, during April 85, during May 102, during June 107, during July 84, during August 111, during Sept. 113, during October 200, during Nov. 237, and during Dec. 276. The entire number throughout the year was 1002; and of this number, there were 469 cases of genuine variola, and 533 of varicella.

Of the 469 patients, 160 had not been vaccinated; in 109 the vaccination, or at least its success, was doubtful; and in the remaining two hundred, the traces of the operation on the arm were obvious and well marked. Of the 160 patients, who had never been vaccinated, 82—rather more than one half—died; of the 109, doubtfully vaccinated patients, 40 died; whereas of the other 200 cases, in which vaccination had unquestionably been performed, there were not more than 25 fatal cases. In six of these 25 cases, the fatal issue seemed to be owing to the putrid character of the accompanying fever; in nine the disease attacked puerperal women, and appeared to be associated with child-bed fever; and in the remaining ten cases, the patients appeared to sink under the violence of the eruptive fever. In all the other cases, the disease ran its course quickly and very mildly;—many of the patients remained almost quite free from fever. The desiccation was sometimes completed in two or three days; and when the crusts fell off, the skin was merely red and tender, but no scars were left behind. In estimating the mortality of the disease in the 200 vaccinated patients, we are to remember that in nine it was complicated with a puerperal affection. In the 533 cases of varicella, there were three deaths; and these occurred in puerperal women.

In conclusion, the reporter insists urgently on the great efficacy of vaccination in mitigating the severity of small-pox, and in checking its dissemination. No one can hesitate for a moment in admitting the truth of this statement, who has had an opportunity of witnessing any of the epidemics of variola, which have of late years prevailed on the Continent. The remark invariably made has been, that the disease has been comparatively mild and innocuous to those patients, who have been unquestionably vaccinated at some former period of their lives; but severe and very fatal to those, who have never undergone the operation. It is worthy of remark that, during the year 1835 in the Vienna Hospital, none of the medical attendants (they were all too wise to have neglected vaccination) exhibited any of the symtoms of the epidemic.—*Mediz. Jahrbuch des Csterr. Staates.*

---

### Case of most obstinate Sternutation cured by a Pinch of Snuff.

A girl 11 years of age was seized on the 18th of November with violent fits of sneezing, which returned so very frequently that there was not more than a quarter of an hour's intermission at a time for the following six weeks. Dr. B. visited her for the first time on the 8th day of January, when he found that the girl's general health was very good, except weak from the want of continuous sleep for so many weeks. She complained of a most intolerable tickling in the nostrils, but, upon examining them, nothing unusual was to be seen.

From this date to the 17th of March a host of remedies were tried without however any permanent relief to this most troublesome symptom. Vapour baths, mustard foot-baths, purgatives, anthelmintics, emetics, leeches to the epigastrium, (against the presumed gastrite,) sulphate of quinine, various forms of antispasmodics, &c. &c. were used in succession. The doctor then thought of the homoiopathic axiom, "similia similibus curantur," and advised his patient to try the effects of a pungent sort of snuff. Three pinches effected a cure!—*Bulletin Med. Belge.*

---

### Remarks on certain Forms of partial Convulsions.

In 1831, M. Dance published in the Archives Generales a memoir entitled "Observations on an intermittent form of Tetanus." The singularity of the phenomena which he had observed in four cases, and the paroxysmal character of the partial cramps or convulsions, had arrested his attention, as indicating a form of diseased action which, he believed, had been overlooked by preceding writers. He was somewhat puzzled to decide, in what point of light he should regard the affection, whether as of a rheumatic, tetanic, or of a purely agueish nature. "We may however," says he, "assert this much, that it has the strongest affinity to tetanus, if we look merely to its phenomena, and to intermittent or remittent fever, if reference be made to its course and progress."

In 1832, M. Tonnelle, ignorant apparently of the observations of M. Dance, communicated to the Gazette Medicale, an interesting paper "on a *new* convulsive disease of children."

Without alluding to the theoretical speculations of M. T. as to the origin of the disease which he has described, we may state that in none of the fatal cases which occurred in his practice, was he able to discover the slightest organic lesion in any part of the nervous system.

The attention of M. Guersent, physician of the Hospital of Sick Children at Paris, appears to have been excited by these memoirs of MM. Dance and Tonnelle; for, in the course of 1832, he also communicated his thoughts on the subject in the pages of the Gazette Medicale. Unfortunately he has associated together every varying form of convulsive contractions; such as those which characterise wry-neck, those which are of a genuine tetanic nature, and lastly, those which accompany meningo-encephalitis.

Dr. W. Murdoch, after having described (Journ. Hebdom.) the chief phenomena which characterise the "spasmodic muscular retractions," (so he calls the affection which we are at present considering,) thus expresses himself:—"We do not know if we can with propriety refer them to any lesion of the centre of innervation, or to a spasm altogether local of the muscular tissue—a spasm which may be compared to a prolonged cramp, or to a partial tetanic stiffness. They cannot well be likened to proper convulsions, because these latter are almost always accompanied with a functional, or pathological alteration in some part or another of the encephalon, nor to chorea, in which the muscular contractions are invariably of the clonic kind."

Dr. Murdoch is inclined to attribute many cases of wry-neck to this muscular affection; although, it must be acknowledged, that the spasm in wry-neck is always progressive and chronic, and the affection is very generally most obstinate of cure—two features, which do not belong to the disease under consideration.

Before entering on any general description of the disease, the narration of a few cases may be deemed useful.

A child, three years of age, of a very irritable constitution, had been affected for a short time with cough, diarrhœa, and evening feverishness, before he was admitted into the hospital. Soon after his admission, the general symptoms having not undergone any change, it was observed that the fingers and thumbs of both hands were affected with a remarkable rigidity: they were bent forcibly in upon the palms, remaining however fixedly separate from each other: the wrists also were bent on the forearms, and these again slightly on the arms. The feet and toes were affected in a similar manner: the gastrocnemii muscles were rigidly contracted, and had drawn the heels forcibly backwards; the toes were bent in upon the soles, and the feet, were directed inwards towards each other.

These novel symptoms had come on rather unobserved, and they did not seem to be associated with any aggravation of the constitutional disturbance. Some days afterwards, the little patient was seized with frequent vomitings; the muscles of the face became occasionally convulsed, and the extremities were affected with clonic convulsions. These symptoms gradually abated, but the tetanic contractions of the hands and feet remained as before: the general distress was unrelieved, the cough being still frequent, the breathing hurried, the pulse rapid, and the bowels relaxed. A few days subsequently, the local cramps ceased altogether, but the constitutional symptoms were decidedly aggravated, and the boy died convulsed at the beginning of the fourth week after his admission. On dissection, there was found slight serous infiltration of the sub-arachnoid cellular tissue, great vascular congestion of the pia mater, and of the cerebral substance: no ramollissement. The spinal marrow and its investing membranes were nearly in the same condition as the contents of the cranium. The right lung exhibited some tuberculous spots, and patches also of lobular induration.

*Remarks.* The progress of this case is interesting. The cramps of the fingers and toes came on without any well-marked signs of encephalic disturbance, and to these symptoms were added afterwards clonic convulsions in different parts of the body. The clonic convulsions subsided, but the tonic spasms remained unabated; and then the latter also ceased spontaneously, although the constitutional symptoms became more aggravated. The clonic convulsions returned at intervals, without any renewal of the rigid cramps, before the death of the patient.

[We have no doubt in our own minds that the preceding case must be regarded as one of genuine hydrocephalus. The practitioner who has seen much of this most insidious and perplexing disease knows well that he is not to look for any uniformity of symptoms, or regularity in their succession. There is no malady which so vexatiously defies nosological definition or accurate description as hydrocephalus.—*Rev.*]

*Case* 2. A child, eighteen months old, had been suffering from cough, diarrhœa, and occasional attacks of clonic convulsions for five weeks, before it was received into the hospital on the 24th of April. At this period there was marked retraction of the flexors of the wrists and fingers of both hands, also of the gastrocnemii, and of the flexors of the toes, and serous infiltration around the wrist and ankle-joints: the pulse was very rapid, and the bowels very frequently relaxed.—"Gomme edulcorée, julep gommeux, cataplasme emollient sur le ventre, bain tiede, lait pour tout aliment," were prescribed. The cramps of the extremities were regarded as the effects of a lesion purely local (!) and on this idea frictions and fomentations were ordered for the limbs. The constitutional symptoms became more and more aggravated, and the child died in convulsions on the first of May. The rigidity of the flexed muscles continued "avec intensité" after death. On dissection the contents of the cranium were found almost quite normal; the tissue of the pia mater was very slightly infiltrated with serum; its vessels only moderately injected; and the ventricles contained a small quantity of limpid serum: the cerebral substance was perhaps rather firmer than usual. The thoracic organs were sound. The mucous coat of the intestinal canal was redder than usual here and there, and the glandulæ Peyeri were somewhat larger and more prominent.

This case affords a very striking example of well marked permanent contraction of the muscles of the fingers and toes without any appreciable lesion in the centres of innervation.

[The details of the case do not satisfy our minds.—*Rev.*]

*Case* 3. A boy, 13 years old, of a healthy constitution, and who had enjoyed good health, with the exception of occasional headaches, which generally relieved themselves by bleeding from the nose, began to experience, while engaged at his work of a type-founder, a sense of numbness in the fingers of both hands, and an involuntary and momentary flexion of both thumbs. These symptoms did not last above a few minutes, and then they ceased entirely: his general health at this time appeared to be quite good. Ten days after the date of their occurrence, he was admitted into the hospital with the following symptoms:—difficulty of deglutition, anorexia, sharp pains in the epigastrium, nausea and vomiting, constipation, cough, evening feverishness, general weakness and languor. He complained also of a numbness in the fingers of both hands, and a retraction of the anterior or flexor muscles of the forearm, which caused a semi-bent position of the fingers and thumbs. There was no distinct symptom of encephalic disturbance. A few ounces of blood were taken by venæsection, but without any abatement of the general feverishness, and the contraction of the fingers had become more forcible, so that they now remained permanently bent in upon the palms, and the thumbs were strongly adducted upon the fingers. Sharp pain was caused by any attempt to overcome the flexion. The integuments of the hands and lower part of the forearms were red, and there was slight puffy swelling around the wrists. Dr. I. ordered "deux pots d'infusion de mauve edulcorée, un julep gommeux, un lavement emollient, un bain, et pour aliment un potage au lait." Very soon after leaving the bath, the fingers of the right hand could be readily extended, and the pulse, which had previously remained quickened, fell to 84. The same treatment was continued for a few days, and all the symptoms, general as well as local, vanished.

*Case* 4. A young man, 18 years of age, began to experience, three months before his admission into the hospital, a marked numbness in his fingers, retraction of the flexor muscles of the right hand, and a sense of restraint and difficulty in moving the forearm. Eight days subsequently, the same symptoms occurred on the left side. In the course, however, of two or three days, all these symptoms disappeared, and the young man was able to resume his occupations. They returned, however, with varying intensity, at intervals lasting usually for four or five days, and then ceasing altogether. He did not suffer from headach; but he felt occasionally a slight vertigo. When received into the hospital, the fingers of both hands were so forcibly bent upon the palms, that they could not be extended; the muscles of the forearms were permanently contracted, and formed a projecting mass on the anterior or volar surfaces, such as is felt on the calves of the legs, when affected with cramp.

He was bled, put upon a low diet, and ordered to have a warm bath. The antiphlogistic regimen was persevered in, and in the course of a week all the symptoms were quite subdued.

*General Reflections.* Dr. Berge, after reporting the preceding four cases, enters upon a critical analysis of the results which they, as well as the cases narrated by Dance, Tonnellé, and Murdoch seem to furnish. It appears from this analysis, that, in the majority of fatal cases in which the tetanic or tonic convulsions of the fingers and toes have been observed, no uniform or constant post-mortem appearances have been discovered in the brain or spinal marrow.

A morbid state of the intestinal mucous lining has been more frequently noticed; and our readers may have observed that, in three of the preceding cases, diarrhœa had preceded the occurrence of the spasms. The sympathy between the bowels and the muscular system is well exemplified in this, as well as in some other diseases, such as cholera, &c.

The occurrence of the spasms has been, in most cases, not quite sudden, but has usually been indicated by certain premonitory symptoms. There is usually, for some days or weeks previously, a feverish state of the system, announced by general weariness, oppression, and pains in all the limbs; the patient experiences, at first, a feeling of numbness, pricking, and tremulousness in the hands, arms, and feet; he cannot move them with ease; they feel stiff and uncomfortable, and often flying pains are felt in the whole extent of the extremities, along the lines of their principal nerves. The contraction of the flexor muscles gradually increases in force, until the fingers are immoveably bent in upon the palms, the thumbs strongly adducted towards the fingers, and the wrists bent upon the forearms.

The feet and legs are similarly affected in most cases; but the lower extremities are, we believe, seldom or never attacked before the upper ones. Sometimes this morbid state of muscular contraction extends to other parts, especially in adult subjects; thus the muscles of the lips, lower jaw, abdomen, and spine, are occasionally attacked. M. Dance observed, in one or two cases, a vibratory agitation of the muscular fasciculi of different muscles, occasioning a distressing pain in the parts affected. The integuments covering the joints were often red, and almost erysipelatous, and sometimes also œdematous —those of the wrists and ankles were most frequently affected in this manner. The tonic convulsions now described were, in many cases, accompanied with or followed by clonic convulsions of some muscles. In no case was strabismus, delirium, or coma observed. The fever which was generally present had the characters of the infantile remittent; the symptoms abating during the day, and becoming much aggravated towards evening. Sometimes it exhibited the form of an almost regular intermittent—so much so, that M. Dance was inclined to view the muscular affection as a form, hitherto unnoticed, of intermittent disease, and induced by miasmatic influence.

The duration of the contractions varied, from a few minutes to several hours, or even days in succession, with only occasional and partial relaxations. In our own experience (says Dr. B.), we have never observed any marked regularity in the return of the attacks. In one case, they continued to return at intervals of greater or shorter extent during a period of nearly four months. Out of twenty-two cases referred to by the author, death occurred in seven. The affection is, therefore, not of so slight a danger as some authors have been led to suppose.

It is generally symptomatic of some grave constitutional disturbance, and is very rarely, if ever, an essential or idiopathic disease. Either the brain, spine, or the intestines are, we suspect, the " fons et origo mali" in almost every case. When connected with encephalic disease, the occurrence of the muscular contractions is very generally preceded by pain in the head, confusion of mind, delirium, disturbance of vision, strabismus, irregularity of the pupil, paralysis, &c. M. Andral, in his Clinique Médicale, relates four cases of ramollissement of the brain, in which irregular contractions of certain sets of muscles formed one of the most prominent symptoms.

It is especially worthy of notice, that in cases of cerebral ramollissement, the convulsions are not unfrequently confined to one side of the body, whereas in other cases, unconnected with cerebral disease, such as have been described by MM. Dance, Tonnellé, and Murdoch, they affect both sides simultaneously; and it may be added, that paralysis is of much more frequent occurrence under the former than under the latter circumstances. In addition to these points of difference, we may likewise remark that, when the brain or the spine are diseased, it is not frequent that the muscles of the extremities only are affected ; the sphincters of the rectum and bladder usually are relaxed, or at least act irregularly at the same time; and the respiration also is almost always more or less disturbed.

When every symptom of cerebral or spinal disease is absent, in cases of morbid muscular contractions, we have strong reasons for suspecting that abdominal irritation is the source of the mischief. Dentition also, in young subjects, is a not unfrequently exciting cause. In reference to the age at which the disease has been observed to occur most frequently, Dr. B. states that, of 22 cases, the particulars of which he has collected, nine occurred in children below five years, five between the fifth and the fifteenth years, and eight in persons above fifteen years.

In conclusion, he enumerates the following characteristic features of the disease—its invading the upper and lower extremities alike, and of both sides of the body at the same time; its attacking the fingers and toes first, and then extending upwards in its progress to the forearms and legs, and to the arms and thighs ; the occasional, nay, rather frequent absence of all cerebral or spinal distress; and, lastly, its short duration and inconsiderable danger in the majority of cases.—*Journal Hebdomadaire.*

*Remarks.*—It is to be regretted that Dr. Berge has not better arranged and elucidated his sentiments on the form of convulsion which he has described in the preceding remarks. After making one extract from the writings of M. Andral, we shall avail ourselves of some remarks of Dr. Constant, published in a recent number of the Bulletin Génerale de Therapeutique.

M. Andral, in his recent course of lectures on the diseases of the nervous system, makes the following remarks on muscular contractions or tonic spasms, as one of the signs of cerebral congestion :—" This sign, consisting in the permanent flexion of the joints—the flexion being in some cases so powerful, that it cannot be overcome by even very strong force—is characteristic of the disease which we are at present considering. These contractions, less constant, and also less decided in the first stage of inflammation, are strong and constant when ramollissement has taken place :" and, in his description of the symptoms of inflammation of the spinal marrow (myelite), he remarks—" In some cases, muscular contractions or spasms form one of its prominent symptoms. The seat of these varies according to the portion of the marrow affected. When they attack the abdominal muscles, they occasion excruciating pain, and often all the symptoms of the most intense colic. Sometimes the cramps affect the muscles of the extremities only ; and these, indeed, are not unfrequently the earliest symptoms observed in some obscurely-marked and inactive cases of the disease, while in other cases, the muscular convulsions have a much more acute character, and simulate very strongly those of chorea, or, as happens in the most aggravated cases, of genuine tetanus. In this last form of the disease, death is almost inevitable."

Dr. Constant thus defines the " maladie convulsive, peu connue et assez commune"—the same, no doubt, as that alluded to by Dr. Berge : " a permanent and involuntary contraction of the muscles of the upper and lower extremities, occasioning a remarkable rigidity of these members, and accompanied or not with pain." The upper and lower extremities are usually affected at the same time ; sometimes, however, the disease is limited to the one or to the other. When the upper extremities are affected, the muscles of the forearms become exceedingly tense, and very prominent ; the wrists are bent ; the fingers are stiff, separated from each other, and inclined to the palms. All movement of these parts is utterly impracticable. The shoulder usually retains its mobility. When the disease affects the lower extremities, the ankle-joints are not, like the wrists, bent upon the legs, but powerfully and painfully extended, while the toes are strongly flexed upon the soles. Sometimes the muscles of the thighs also suffer. In one case seen by Dr. Constant, the adductor muscles were so predominant, that the limbs were immoveably crossed, the one upon the other. While the muscular system is so seriously affected, the mental and sensorial functions remain untouched ; the pulse is but little affected, except when the pains are severe. It is rare that the muscles of any other parts than the extremities are affected with spasm.

The duration of this convulsive disease varies from a few hours to several days, weeks, or even months. Sometimes it is continuous—at other times it is intermittent. Occasionally it alternates with regularly clonic convulsions ; and in several cases it has seemed to cease spontaneously. Twice have we seen it cease on the appearance of the catamenial discharge, and twice on the eruption of measles. When uncomplicated with cerebral disease, it seldom proves fatal. Dissection has hitherto failed in detecting any uniform organic cause of the disease.

The first case, related by Dr. C. occurred in a child a year and a half old, who had never well recovered from the measles. The spasms of the upper and lower extremities came on very suddenly, and was no doubt attended with great pain, as the child kept incessantly crying. It seemed, however, to be quite sensible. It died in the course of ten hours. On dissection, no distinct traces of disease were found in any part of the nervous system. The pleura and lungs were inflamed in this, and also in other three similar cases. "Dans tous les cas," adds the author, "les centres nerveux et leurs annexes ont été trouvés exempts d'alteration."

In the second case, the spasms of the extremities supervened on an obstinate diarrhœa. The spasms lasted, with only partial relaxation occurring occasionally, for nearly three weeks before death. An attack of general convulsions preceded the fatal event.

The only morbid appearances found on dissection in the encephalon was a trifling serous exudation under the arachnoid coat: the cerebral substance was pale, but its consistence was normal.

In some cases teething appears to have some influence in inducing this spasmodic affection. Worms also, and indeed any irritation of the intestinal tube (as existed in the last case, and such as is present in all severe cases of cholera), may bring it on.

In more advanced years the changes which accompany the development of the sexual organs, the first appearance of menstruation, sudden mental emotions, &c. are occasionally accompanied or followed by a spasmodic affection of the extremities. When the disease is associated with febrile irritation, and with any disturbance of the mental and sensorial functions, we have reason to suspect cerebral disease; especially ramollissement of the brain.

The treatment of this convulsive affection must be modified by a variety of circumstances; such as its probable primary cause; the age of the patient; the existence of previous disease, &c. When there are no symptoms of cerebral disease present, warm-baths, purgatives, carminatives, antispasmodics, and the use of external irritants to some part or another of the spine will be found to be the most efficacious remedies. The state of the bowels ought to be in all cases minutely examined and attended to. Sometimes the spasmodic affection appears to be induced, or at least to be much promoted by a low and exhausted state of the system, giving rise to an inordinate irritability of the nerves. Warm aperients, sedatives, tonics, and a generous light diet, will be proper under these circumstances."

We deem it worthy of mention here that the treatment which in very numerous cases of partial, as well as of general convulsions occurring in children has been successfully pursued by us, consists in the use of a few very simple remedies.

The practitioner must first of all endeavour to ascertain whether the convulsions proceed from organic cerebral mischief, or from mere irritation in the head, teeth, bowels or kidneys. The state of all these parts is therefore to be sedulously examined. If there are any convulsive movements of the eyeballs, if the pupils are permanently contracted or dilated, if there is any difficulty in swallowing, if the pulse be very rapid, and if there is a tendency to stupor and oppression, there is much reason to suspect an unfavorable termination of the case from cerebral disease. On the other hand, if the attacks of convulsion are succeeded by intervals of almost complete ease and repose, if the child screams repeatedly and grinds its gums, if these feel to the finger hot, swollen and tense, we should not for a moment hesitate to relieve them by a free use of the scarificator, and to induce a derivation from the encephalon by means of active diuretic purges.

When irritation of the bowels or kidneys is the exciting cause of convulsions, the attention of the enquiring physician is usually excited, by discovering that the little patient is every now and then seized with attacks of pain, aggravated before and during the urinary or alvine evacuations; that the physical qualities of these evacuations are not healthy; that the abdominal muscles are every now and then contracted and drawn into lumps, and that the symptoms, which we have described as peculiarly indicative of cerebral and of dental disturbance, are absent or only indistinctly present.

A dose or two of calomel with carbonate of soda, followed by the free use of the infusum sennæ combined with manna and tartrate of soda, and by the exhibition of a senna enema (never to be neglected) will very generally afford quick and decided relief.

In cases of more chronic convulsion, when the origin of the disease is obscure, and the leading functions of the system do not appear to be very obviously disturbed, we have derived great advantage in numerous cases from a steady use of the following treatment. A powder consisting of hydrargyrum c̄ creta, pulv. rhei and sodæ carbonas, is to be given every evening; and during the day, small doses of liquor potassæ and vinum ipecacuan in any distilled aromatic water. In some cases, a small blister behind one of the ears, or the friction of antimonial ointment on the nape of the neck, may be beneficially employed. The use of purgative enemata every second or third day ought never to be omitted. They are among the most direct derivatives in all cephalic diseases. The great and leading principle to guide us in all cases, is to bring and keep the alvine and urinary secretions in a healthy state, and to relieve any local irritation.

It is not by following any one exclusive mode of treatment, or the directions of any one teacher, that we can succeed in rightly understanding and in successfully opposing so Protean a disease as convulsions. A sedulous enquiry, minute examination, a quick and unprejudiced observation, and a simple and decided mode of treatment—these are the necessary implements for the practical physician.—*Rev.*

---

### SEVERE SYMPOMS FROM THE BITE OF A SPIDER.

A young girl, gleaning in the fields, was bit above the left bosom by a large dark-coloured spider. She felt a sharp pain in the part at the time; and in the course of a few minutes she became so weak that her limbs sunk under her, and her sufferings were so great that she rolled about on the ground, and could not refrain from screaming out. When Dr. Hameau visited her in about an hour after the accident, he found her drenched with perspiration; her face alternately pale and flushed, her extremities cold, the breathing slow and oppressed, and the pulse irregular and very small. She complained of severe pains in the feet, knees, thighs, and in the small of the back; and as these subsided, they fixed themselves in the epigastric region, causing a sense of most distressing oppression and anxiety. The muscles in several parts of the body were in a state of continual oscillation, or tremor: firm compression of the limbs afforded considerable relief to this symptom. The seat of the bite was red and swollen, and a small vesicle filled with a yellowish serum occupied its centre.

Dr. H. opened this vesicle and moistened the part with some fluid ammonia, and ordered the patient to have a warm-bath and a powerful opiate. The symptoms however were not relieved, until three doses of opium had been exhibited. The pains then abated and a general reaction came on. Towards evening however, the symptoms were partially renewed, and three grains more of opium were given. No narcotism was induced. On the following day, the patient was altogether much better. Some degree of salivation, and also a papular eruption made their appearance; but these symptoms quickly ceased, and the girl was soon quite well.

*Remarks.* It is probable that the bites of all spiders are more or less poisonous. That they kill their prey by inserting a poison into the wound which they inflict, is generally admitted.

Whether the spider in the preceding case was of the tarantula tribe of araneæ we cannot determine, in consequence of the want of any accurate description of its appearance. This spider, the most formidable of its genus, is occasionally met with in the South of France, but much more frequently in Italy and some other warmer regions. No doubt many of the tales of tarantulism,—characterised, we are told, by an irrestrainable propensity to dancing—are fabulous, or at least much exaggerated; but that the bite of some of the larger spiders may induce certain spasmodic, or convulsive symptoms, like those of chorea, is far from being improbable.

Travellers tell us of spiders in the hot climates, as in Guinea, Cape of Good Hope, Madagascar, Ceylon, &c. whose bites have proved fatal. We cannot vouch for the correctness of these reports.—*Bulletin Medicale de Bordeaux.*

---

### Poisoning from a Viper-bite—Cure with Sulphate of Quinine.

A countyman, 44 years of age, was bit by a viper between the fore-finger and thumb of the right hand. On the following day, when admitted into the hospital, the whole extent of the right arm was enormously swollen, and its surface was of a livid red colour. The face and trunk had a jaundiced hue, and the extremities exhibited diffused patches of redness. There was great prostration of all the vital powers; the pulse was scarcely perceptible; the body was bedewed with a cold clammy sweat; the face was convulsed, the pupils dilated, the breathing laborious, and there were frequent efforts of vomiting. Dr. Butazzi, calling to mind the results of some cases recently published in the Filiatre and in the Révue Med., prescribed quinine in large doses, three grains to be taken in a spoonful of wine every hour.

On the following day, the arm remained as much swollen as before, the pulse was scarcely to be felt, and the patient complained of pain in every part of his frame: the dose of the quinine was increased to four grains. On the next day, there was a decided amendment; a copious warm perspiration had taken place, the discoloration of the surface abated, and the features were recovering the character of health. The swelling of the affected arm gradually from this time subsided, and, under the use of oily embrocations, altogether disappeared. Two drachms in all of the quinine were taken in the course of between two and three days.—*Filiatre di Napoli.*

---

### Remarks on the Frequency of Gangrene of the Lungs in the Insane. By M. Guislain.

Laennec mentions that he had not met with more than six or eight cases of genuine gangrene of the lungs in the whole course of his practice. It was in the year 1824, when dissecting the body of an insane patient, that I first observed a peculiar degeneration of the lungs, which I have subsequently had numerous opportunities of observing. That there is a certain sympathy or alliance between mental disorders, and the diseases of the respiratory organs, was noticed long ago by Mead; and the truth of the remark has been confirmed by the researches of Esquirol and others. But I am not aware that any writer, with the exception, perhaps of M. Calmeil (who, in an article in the Dictionnaire de Medécine, has alluded to having witnessed two cases), has pointed out the not unfrequent occurrence of gangrene of the lungs in the bodies of lunatic patients. All that relates to the symptoms, and more especially the causes of this disease, are involved in much obscurity. Laennec was of opinion, that gangrene of the pulmonary texture is very generally attributable to some specific or essential influences, similar perhaps to what occasion carbuncle and pustula maligna, and that rarely indeed is it ever owing to a preceding inflammatory action. Our own experience agrees so far with this statement of M. Laennec, that we fully believe that pulmonary gangrene, occurring in the insane, may be usually traced to a depravation of the blood and other fluids of the body, induced by some disorder of the digestive and assimilative functions. It is well known that some lunatics will refuse for a great length of time to eat any food, or that they will devour the most unwholesome and indigestible articles. We need not, therefore, wonder, that a humoral dyscracy is induced; but why, in this state, the lungs should more especially be affected, it is not easy to discover.

In the first case which fell under my notice, my attention was drawn to the state of the lungs by the unusual offensive smell which was given out when the thorax was opened.

Soon after the occurrence of this case, I met with a patient, whose breath emitted a similar odour; and in whom I predicted that the lungs had become gangrenous. My prediction was verified on dissection of the body; the lungs were quite sphacelated. It ought to be mentioned that the patient had, for a length of time, refused to take any description of food. From this period, I was led to believe that there was some relation, perhaps of cause and effect, between the lesion of the digestive functions on the one hand, and the decomposition of the pulmonary tissue, and the offensive state of the breath, on the other.

The mere mephitism of the inspired air is not, however, an unequivocal sign of pulmonary gangrene; for this phenomenon is often observed, to a marked degree, in cases of protracted abstinence, and also, it is well known, in suppuration of the lungs; but the odour of gangrenous degeneration is certainly peculiar and characteristic. The refusal of food, which I take to be in most cases the remote or inducing cause of pulmonary gangrene, is not an unfrequent symptom of melancholia. This morbid symptom occurs, to a greater or less extent, in almost one-ninth of the entire number of lunatics. Often no means of persuasion will succeed in overcoming the patient's aversion from all food. They will live for twenty, thirty, nay even for sixty days, without any food, except only cold water for drink. Some lunatics will refuse food for some days of the week, and, on the other days, they will willingly eat whatever is given them; in others, the degree of aversion is proportional to the disturbance of the mental energies.

It has been supposed that this unnatural abstinence must be always the result either of some hallucination or delusion of the mind, leading the poor patient to suppose that his food has been poisoned, or of a preconcerted design to put an end to his existence. These influences may operate in some cases, but certainly they do not in all; and we are quite satisfied that the repugnance against all sustenance often arises from a strange perversion of the will, over which the patient has no control, and of which he has no consciousness. Hitherto, we have not observed any facts which might lead us to suppose that this extreme anorexia is ever occasioned by any primary or idiopathic malady of the digestive apparatus.

It is almost unnecessary to state, that a protracted abstinence from nourishment must induce an unhealthy state of the animal fluids. It is to this humoral irregularity, or dyscrasy, that we attribute, as already noticed, the occurrence of pulmonary gangrene in the insane. In periods of religious fastings, some persons, who are rigid in the observance of these, are always more or less affected with cough, and an affection of the breathing. I have for some years past been in the habit of attending many of the members of a sect, which is unusually austere in all its penances. Animal food is interdicted among them, and every now and then a rigid fast is exacted. Often have I had occasion to observe troublesome coughs, dyspnœa, and even hæmoptysis, induced in the more delicate of these devotees. Fortunately, in most cases, the troublesome symptoms were readily curable by the use of a generous diet, and of tonic medicines.

Diseases of the chest are frequent in prisons, workhouses, and other establishments, where the diet is poor and insufficient. It is to the long privation of nutritious food, coupled indeed with the large sanguineous depletions, that we may often trace the teasing cough which occurs during the convalescence from acute diseases, and which is best remedied by a cautious administration of a generous diet. Over-prolonged suckling, masturbation, and, indeed, whatever induces or is accompanied by systemic weakness, such as rapid growth at the period of puberty, will often induce symptoms of pulmonary disturbance.

From all these considerations, I infer that protracted abstinence, such as we sometimes meet with in lunatics, must necessarily have a directly hurtful effect on the respiratory organs. I shall now adduce a few illustrative examples.

*Case* 1. A middle-aged female was so alarmed during "nos derniers commotions politiques," that she lost her reason almost completely. For several successive days she refused all food. Her physician prescribed the repeated application of leeches to the epigastrium. Her friends, being alarmed at her weakness, emaciation, and mental derangement, had her conveyed to our establishment in February, 1831. The only food which she had taken during the previous four weeks was "soupe au lait" and "bouillon leger," in small quantities. Although emaciated, and exhibiting all the appearances of a humoral cachexia, she still retained considerable strength, so as to resist all attempts to make her eat. Her breath began to emit a most offensive odour; the sputa were of a brown rusty colour, in large quantities, and apparently sanious. She died in a state of extreme emaciation.

On dissection, the encephalic and abdominal viscera were free from any obvious alteration of structure. The thorax being opened, the lungs were found to be so soft and friable, that, in attempting merely to raise the left lobes, my fingers sunk into their substance. The stench was so intolerable, that I was obliged to desist from the examination for a short time. When the lung was removed from the thorax, the posterior surface of its upper lobe was discovered to be almost black, and spotted over with greenish and brown patches. On cutting into its substance, the pulmonary tissue was seen in a state of gangrenous decomposition; a dark-coloured offensive sanies, mixed with flocculi of pus, flowed out, and on scraping the lung with the scalpel, the blade was covered with a putrid brownish matter. Nearly one-half of the left lung was in the diseased state now described. The bronchi were filled with a frothy, reddish, fetid mucus. The right lung was not much affected.

It is almost unnecessary to narrate the particulars of the other cases which I have met with. I shall, therefore, merely mention the more important phenomena which they presented. Out of thirteen insane patients who died from inanition, in nine were the lungs found to be gangrenous. It is to be observed, however, that in no case were the two lungs seriously affected at the same time; the lung of one side was invariably more affected than that of the other. In seven of the nine cases, the disease was confined, or nearly so, to the left lung. M. Calmeil, in speaking of the pulmonary diseases to which lunatics are subject, has remarked that the left lung is much more frequently diseased than the right one. The degenerated portion is always circumscribed, never exceeding a fourth or fifth part of the entire lung, of a dark colour, soft and pasty to the touch, and insufferably stinking when lacerated or divided. In three of the remaining four cases, the lung did not exhibit any decided alteration of texture; only its colour was much deeper than usual. In the other case, there was found a sanguineous engorgement or ecchymosis on the posterior surface of the lung.

The gangrenous affection was in all the cases more conspicuous towards the apex, than towards the base of the organ; and in all, with the exception of one only, was it situated in its posterior part.

In almost every case, purulent flocculi were found in different parts of the diseased mass; but the quantity of pus was too small, for any one to suppose that it was owing to a previous inflammatory action; and, moreover, the gangrenous portion of the lung did not exhibit any appearances of having been so affected. The pulmonic texture immediately surrounding the gangrenous part was dark, and looked as if it was ecchymosed.

In all the cases, the patients did not seem to suffer any pain in the chest; there was little or no cough, nor dyspnœa; the system was not feverish, and the pulse was sometimes slower and more oppressed than it had been before. In M. Andral's work on the Diseases of the Encephalon, there is an instructive example of gangrene of the lungs, occurring in a starved woman, recorded.

The poor wretch was admitted into La Charité in the most emaciated condition. She did not, however, suffer from cough, nor from difficulty of breathing, although a portion of the right lung, towards its summit, was found on dissection to be as black as ink, and to be converted into a "putrilage," which emitted an intolerable stench when exposed. There was an organic disease of the brain.

In all the cases of inanition occurring in insane patients, which have occurred in the practice of Dr. Guislain, there was a peculiar and a very characteristic discoloration of the skin during life. This discoloration is usually observed on the face—at first of a reddish or brick hue, and gradually becoming more and more brown or purplish. The eyes are surrounded with brown areolæ; the apex of the nose and lips are livid, and the whole complexion is a blueish earthy hue. A criminal, who recently starved himself to death, presented, in a very marked degree, a cyanotic discoloration of the whole surface of the the body. It is probable that it is owing to the altered condition of the blood, induced by the defective supply of chyle.

It is not easy to determine the reason why the lungs are more subject to a gangrenous affection under these circumstances than any of the other viscera. The lungs, it may be worth noticing, are the important organs of sanguification; but, whether this consideration can lead to any rational conjecture as to the cause of the gangrene, is indeed very doubtful. We have already alluded to the great frequency of pulmonic diseases in insane persons. According to the report of M. Georget, more than one-half of the inmates of the Salpetrière die from diseases of the chest. Many suffer from asthma, chronic catarrh, latent inflammatory attacks, terminating in suppuration, &c.; but these affections are very different from the gangrenous affection described above. They are accompanied with symptoms, more or less obvious and well-marked, of respiratory distress; there is cough, shortness of the breath, hectic fever, night sweats; and, on dissection, we find adhesions of the pleuræ, portions of the lungs hepatized, or infiltrated with purulent matter, &c.

But, in the spontaneous gangrene of the lungs, the symptoms during life are much more obscure. The breath certainly has sometimes a truly putrid smell, and the peculiar wasting of the entire frame, and the discoloration of the surface, are very generally, nay always present. Cases, however, occur, in which the disease has not been even suspected, but where a considerable portion of the lungs is found, after death, in a state of thorough gangrenous decomposition.

In none of the cases examined by Dr. G. did the stomach exhibit any signs of inflammation, or other morbid action. This feature of the disease deserves notice, as suggesting a difference between the physical effects of *voluntary* and of *involuntary* inanition. The experiments and observations of Haller, Magendie, and others, satisfactorily demonstrate that protracted hunger, in man and in the lower animals, always induces some lesion of the stomach, which is discoverable on post-mortem examination, as inflammatory congestion, and even erosion of its mucous coat. Not so, however, with lunatics, who perish from an involuntary inanition. That they experience no cravings of hunger, is proved by the statements of those who fortunately recover from this sito-phobia. They feel no pain in the epigastrium, nor thirst, nor is there any reaction of the sanguiferous system, indicated by a frequency of the pulse and heat of the skin, as is usually observed in cases of voluntary protracted fasting. The loss of strength, too, is never so speedy, nor so apparent, as when the person suffers from hunger. In some of the patients whom Dr. Guislain examined, the corporeal energies were retained in a remarkable degree, even after fasting had continued for several successive weeks; whereas the deprivation of food from a sane person generally proves fatal in from eight to fifteen days.

The miserable sensation of continued hunger is always followed by a febrile irritation of the whole system, which accelerates the approach of death. Dr. G. is inclined to suppose that in some cases, at least, of the aversion to food in lunatics, not only is the sense of hunger quite extinguished, but that even the stomach loses its power of digestion; for he has observed that when food has been swallowed, it has either remained quite unchanged, or it has undergone a decomposition which was evidently the result of mere decay, or putrefaction.

The etiology of that singular complication of diseases—the entire loss of appetite for food, and the gangrenous affection of the lungs—is ingeniously explained by Dr. G. in the following manner.

He supposes that it proceeds from a peculiar apathetic state of the nervi vagi—those nerves on which the sensation of the "besoin de respirer," and of hunger more immediately depend. That the nerves of general or common sensibility are often extraordinarily torpid in insanity is well known to every one, and is amply demonstrated by the insusceptibility of the lunatic to the impressions of cold, heat and pain. To an analogous state of the nervi vagi we may with some probability attribute the absence of hunger, of all gastric irritation, of cough and of any pulmonic distress, which so signally characterise the disease which we have been considering.

It is an admitted fact in pathology, that when the nerves of any part are affected with torpor, there is a tendency to sanguineous congestion in it. We believe that the gangrene of the lungs, such as we have described, is always preceded by an accumulation in and partial obstruction of the blood through the pulmonic texture.

The beautiful experiments of Dr. Brachet have thrown considerable light on this topic, and we are of opinion that the observations which we have made in some of our cases tend to illustrate and confirm the correctness of his speculations on the functions of the nervi vagi. According to Brachet they are not only the conductors of the sensation of hunger, but they also convey to the brain the feeling of repletion of the stomach. The contractile powers of the stomach depend on these nerves, while the ganglionic nerves preside over the functions of the exhalation and secretion of the gastric juices. It is the eighth cerebral nerves which, independently of the "besoin de respirer," communicate to the brain the sense of uneasiness which is occasioned by the lodgment of mucus or of other matters in the air-passages; and it is by virtue of its energies that expectoration or vomiting is induced to remove the offending cause. Whether the nervi vagi exercise any direct influence on the actions of the heart is doubtful; some suppose that they do, while others account for any such *apparent* influence, by attributing it to the co-existent disturbance of the respiratory organs.—*Gazette Medicale.*

---

### Injections of Gaseous Chlorine in Hydrocele — Treatment of Chilblains.

We are informed that Dr. Deblois of Tournay has treated successfully a great many cases of hydrocele, by using chlorine as an injection into the vaginal sac in lieu of wine, or other stimulating fluids. We are not aware that any trial of this remedy has been made in this country, and as we are told that it has answered admirably well in the practice of Dr. D. it may deserve to be introduced to the notice of the English surgeons. The gas is to be permitted to remain for two or three minutes in the sac; and if sufficient irritation is not induced by the first dose, a second or third insufflation is to be practised. One advantage which it is said to have over the injections in ordinary use is, that the gas penetrates into the sac more uniformly, and irritates equally every part with which it comes in contact. The narrator recommends the use of these gaseous injections in that form of goitre, which has of late years been called hydrocele of the neck.

In concluding his remarks on chlorine, he praises in high terms a lotion composed of one part of liquid chloride of lime and five of water as a very effectual remedy against chilblains, whether they are ulcerated or not. The affected part is to be kept constantly wetted with the lotion.—*Bulletin Med. Belg.*

---

### Cure of an Obstinate Ozœna.

A girl, 16 years of age, of a marked scrofulous constitution, and who was very subject to impetiginous eruptions, had laboured under a most offensive ozœna for upwards of four years. It had come on originally without any appreciable cause, and had been subjected to various modes of treatment. Leeches, poultices, local vapour-baths, sulphureous as well as simple, blisters to the neck, frictions with mercurial ointment, injections of various substances, more particularly of the chlorides, had been tried successively, without however affording any decided relief.

A very eminent surgeon, consulted in 1834, recommended the application of blisters to the nape of the neck, the frequent insufflation of a powder consisting of equal parts of calomel and of pulverised gum arabic, and the injection of a lotion containing the oxymuriate of mercury and opium in solution. The use of these remedies had been persevered in for upwards of four months with only a temporary benefit. When she consulted Dr. D. in November 1834, the following is the report of her case. The nose, and more particularly the left ala, was red, inflamed, and immensely swollen; the edges of the nostrils were excoriated and painful. The patient complained of an intolerable itching in both nostrils, and a sense of tension over the frontal sinuses. There was a constant discharge of horribly offensive mucus from both nostrils. Six leeches were applied over the root of the nose and the frontal sinuses, and the use of a mild vegetable regimen recommended.

The irritation having abated, a course of iodine was commenced and continued for four months. This however failing, Dr. D. says he was not a little puzzled what to suggest. Fortunately he thought of inserting a seton in the nape of the neck. No sooner had it begun to discharge than the condition of the nose evidently mended, and in the course of a month, the running from the nostrils, and the tension of the forehead had almost quite subsided.

The seton was kept open for other five weeks, and the cure of the ozœna was complete and permanent.—*Ibid.*

There is a long article in one of the recent French journals on ozœna, or "punaisie," as it is called, recommending strongly the direct application of the nitrate of silver as the most effectual remedy.

No doubt it is a useful application; but we are convinced that most cases of this disease are connected with a depraved state of the general health, and that it is only by ameliorating this, that we can hope to cure obstinate ozœnas. The internal exhibition of the muriate of mercury, and of the hydriodate of potass, have had a marked beneficial effect in some instances, where venereal taint was not suspected. —*Rev.*

---

### On the Coincidence of Endocarditis and of Pericarditis with Acute Rheumatism.

Professor Bouillaud informs us that, during the last three years and a half, his attention has been directed in an especial manner to the examination of this most important pathological position, that in nearly one-half of the patients suffering from acute rheumatism whom he has attended, inflammation of the sero-fibrous tissues of the heart was discoverable.

He has been led to this discovery (for like most of his countrymen he *re-discovers* many a well-known fact) by ausculting the sounds of the heart in every case of acute rheumatism under his care, and finding that in a majority of these the abnormal blowing, rasping or sawing sounds were as distinctly audible as in most instances of old organic disease of the heart, although the patients had never suffered from any cardiac distress previous to the invasion of their rheumatic attack.

From a most extensive experience he is now quite convinced that an acute affection of the heart in cases of active pyrexial rheumatism is to be regarded not as a mere accident, or a rare and fortuitous complication, but as one of the most frequent accompaniments of the disease. Many of our medical brethren have, he says, reproached us with exaggeration on this point, and they have most discourteously alleged that either we have often *imagined* the existence of pericarditis and endocarditis, when no such lesions existed, or that the great frequency of these cardiac affections in our practice of late must be attributable in a great degree to what has been denominated a "medical constitution of the atmosphere."

Professor Chomel has in the last resumé of his clinique adduced 49 accurately observed cases of acute rheumatism, where no cardiac complication was, he says, discoverable. Now these very cases seem to have occurred to him at the very time, when many of our's were under treatment; and the difference alluded to between these two sets of patients must therefore be attributable less to any difference in the medical constitution of the season, than to a difference in the diagnostic abilities of M. Chomel and of myself. We have made a point of examining attentively the chests of all our patients; M. Chomel and others have probably neglected to do so:—"pour trouver il faut chercher, et chercher avec un grand soin." The existence of pericarditis in a rheumatic patient is announced by the following symptoms: dulness on percussion of the precordial region over a greater extent than in health, a certain degree of fulness or swelling there, the pulsations of the heart being less distinctly felt by the hand, the sounds on auscultation being obscure, more distant, as it were, from the ear of the observer, and also being accompanied with various abnormal bruits, some of which depend on the friction between the two opposed surfaces of the pericardium, and others on the complication of endocarditis with pericarditis. Pain more or less acute over the heart, palpitations and irregularities of the pulse are in many cases associated with the preceding symptoms. The abnormal bruits accompanying the double action of the heart, and the irregularities of the pulse, accompanied frequently with a "frémissement vibratoire," are more decidedly marked in endocarditis, than in common pericarditis, while the increased extent of the dulness in percusssion, and the more or less prominent swelling of the precordial region are more conspicuous in the latter than in the former affection.

Some writers have objected to the doctrine of the frequent co-existence of cardiac inflammation with acute rheumatism, on the ground that the former disease is of grave and most dangerous import, whereas acute rheumatism very rarely indeed terminates fatally. But the data for this reasoning are far from being quite correct. Louis among others has shewn that the danger of pericarditis has been greatly exaggerated, and the error on this point is no doubt attributable to the disease having been very often overlooked, when the patients recovered. This doctrine is amply confirmed by the results of our own experience.

During the last clinical course numerous cases of acute pyrexial rheumatism were treated at La Charité, and the data which they afforded have most undeniably proved that "in a large majority of cases of 'rheumatisme articulaire aigu *generalisé*,' the coincidence of a cardiac affection was the rule, and the non-coincidence was the exception." Such being the case, it is scarcely necessary to enter on the consideration of the appropriate treatment of acute rheumatism. Large and repeated bloodlettings, the application of leeches or of the cupping-glasses, the internal use of calomel with opium, of colchicum, &c. are the only safe and efficacious remedies.—*Journ. Hebdom.*

Our readers will find similar views professed and explained in the review of Bouillaud on Diseases of the Heart, published in the last number but one of this Journal.—*Rev.*

### Cases of Glanders in the Human Subject.

An ostler in the royal stables at Berlin, a healthy robust man, was seized on the 4th of October with attacks of shivering, and with general indisposition. He continued however to attend to his work until the 9th, on which day he suffered so much from severe pains in his back and limbs, that he could not leave his bed. On the following day he complained of pains through the chest, difficulty of breathing and cough; and these symptoms were accompanied with considerable febrile irritation. The thoracic symptoms were relieved by the adoption of antiphlogistic remedies, but the fever was unabated, and the patient's strength was becoming more and more impaired. He was admitted into the hospital on the 18th of the month. The prominent symptoms were severe pains in the left thigh and leg, (the inner surface of which exhibited patches of erysipelatous inflammation), and also in both upper extremities, along the back, and in the parietes of the thorax. The pulse was rapid, full and soft, the tongue was not much furred, but the appetite was quite deficient. As the disease resembled a rheumatic fever, the treatment consisted in the use of warm baths, saline diaphoretics, and of a blister applied to the chest.

In the course of the following night, the patient became delirious, and the pulse was small and feeble. Leeches and cold lotions were applied to the head; but without mitigating the symptoms.

On the 21st numerous boils, and dark coloured inflamed patches were observed on different parts of the surface. The largest of these was situated on the right cheek; it was nearly three inches long, and its appearance indicated a tendency to gangrenous changes. The patient lay quiet, oppressed, but not insensible; his pulse was very rapid, small and weak, and the skin was bedewed with copious perspiration. On the following day, a fresh crop of boils appeared, and the patient was repeatedly delirious.

On the 23rd the stupor was more decided; the pulse was more rapid than ever; the breathing was much distressed; and, as the fatal event approached, subsultus tendinum, and convulsions alternated with a state of profound coma.

*Dissection.* A distinct fluctuation was perceptible in many of the boils, and in the intervals between some of these were observed numerous insulated pustules, not unlike to those produced by the vaccine virus, filled with a yellow matter. An incision into the inflamed spot on the inner surface of the left leg opened a considerable sized abscess, which contained five or six ounces of unhealthy pus. The examination of the boils proved that they were genuine abscesses, which had their seat in the substance of the muscles. The texture of both lungs towards their apices was softened. On the inner surface of the lower lobe of the right lung was found an abscess, which corresponded with, (uebereinstimmte) with an exterior one situated in the intercostal muscles. The head was not examined.

(It is much to be regretted that the details of this dissection are so scanty and badly reported.)

*Case* 2. A man, 49 years of age, having had the charge of some glandered horses, became affected with shiverings, sense of confusion in the head, loss of appetite, dragging pains in the back and limbs, and universal debility. These symptoms had continued for several days, before an erysipelatous inflammation made its appearance on the inner side of the right calf, which was hot, swollen and painful. The pulse was frequent and soft; the respiration was considerably hurried; and the thirst was very urgent. In the course of the night, the patient was delirious for several hours. On the following day, numerous boils appeared on both upper extremities, and also on the thorax. (Infus. rad. angelicæ cum tinct. valerian æthereâ). The patient died comatose.

*Dissection.* The boils were found to be genuine abscesses, seated in the muscular substance. An incision into the inflamed spot on the right leg discovered a large purulent sac, which contained upwards of six ounces of offensive unhealthy matter.

On opening the cranium, a lymphatic exudation between the pia mater and the arachnoid membrane was observed; a considerable quantity of serum was in the lateral ventricles; and the veins of the cerebral substance were congested with dark blood. In several spots on the anterior surface of the lungs were observed numerous pustules, similar in appearance to those on the skin. In the pulmonary parenchyma there were several indurated and knotty inflamed points; but no traces of pus were detected in these.

*Case* 3. A youth, 20 years of age, was admitted on the 8th of July into the hospital with the following symptoms. He complained of sharp dragging pains in the extremities and along the spine, of great prostration of strength, &c. On the following morning was observed an erysipelatous blush on the inner side of the left knee-joint, which when moved, or even merely touched, was exceedingly painful. In the course of the day, another rosy patch made its appearance over the head of the fibula. It was of a dark red colour, and very tender on pressure. The patient reported that his indisposition commenced on the 27th ult. and that the severe rheumatic pains set in on the 2nd of the present month. Several doses of an antimonial emetic were administered; warm baths (for a full hour at a time) were used, and the affected knee was repeatedly leeched. In the muscles of both extremities were perceived numerous tubercles or knotty points, which were very distinct to the finger passed along. Between these points were situated small, insulated pustules, filled with a yellowish matter, and surrounded with an erysipelatous border.

No satisfactory account of the origin of his illness could be obtained from the patient himself; but his father states that his son had had charge of a glandered horse for several weeks, and that he had once or twice slept in the stable. On the 11th, numerous fresh boils made their appearance on the face and the extremities. The tongue was parched and invested with a brown crust; the pulse was rapid, small and weak. (Infus. Rad. Serpentariæ cum Liq. Ammon. Succin., Liquor. Ammon. Acet. and Æther. Sulphuric.)

On the left knee a large discoloured vesicle arose; when punctured, a considerable quantity of dark offensive discharge flowed out. The number of pustules gradually increased; several appeared on the prepuce and glans penis. An ichorous slime flowed from the nostrils and from between the eyelids. The patient became delirious and comatose, and death supervened during the night of the 12th instant.

*Dissection.* The abscesses in the muscles of the extremities and of the trunk were very similar to those observed in the preceding two cases. They were seated in the muscular texture, and when opened, were found to contain genuine pus. The largest were found imbedded in the biceps brachii and other muscles of the arm; the smallest were those situated on the dorsum of the foot. The erysipelatous spot on the left knee-joint had passed into a state of genuine gangrene.

From the history of these three cases, it appears that this most fatal disease commences with all the ordinary symptoms of rheumatic fever. The first stage lasts usually for ten or fourteen days, and may be viewed as a "stadium ebullitionis" to the second stage, which is characterised by an eruption of cutaneous pustules, and of muscular abscesses, under the form of furuncles or boils, and by the supervention of typhoid symptoms, delirium, stupor, and tendency to gangrenous changes of the affected parts.

The prognosis in all cases is invariably most gloomy. Few or no cases of glanders-infection have recovered.—*Med. Zeitung fur Heilk. in Preussen.*

In one of the numbers of Hufeland's Journal for last year, two cases of glanders-infection in the human subject are recorded.

A middle-aged man, who had been exposed to the infection from having to take charge of a glandered horse, began to ail on the 7th of April. A week afterwards, when admitted into the hospital, he complained of pains in all his limbs and along the spine; and on his arms and legs there were observed several small tumors, unequal and elastic to the touch, and retaining the natural colour of the skin. Mercurial ointment was rubbed upon these; some of them diminished and almost disappeared; one or two advanced slowly to imperfect suppuration. The patient gradually became hectic, was distressed with cough and obstinate diarrhœa, and died in the second week of the following August. [The course of this case was much more protracted than in most cases of glanders-infection.]

*Dissection.* The abscesses contained a viscid and unhealthy pus; the surrounding cellular tissue was destroyed, and the substance of the adjacent muscles was degenerated and wasted. The lungs were diseased in various parts; being either hepatized, tuberculated, or softened and decayed. The trachea was inflamed—the intestines were normal. [A most unsatisfactory narrative.]

In the next case, the disease (if it was really glanders), was still more slow and tedious in its fatal progress. A young soldier, who had for some weeks been occupied in the stables where there were several glandered horses, was attacked with feverish symptoms, which were accompanied with pains over all his body and limbs, and with sharp stitches in the side. These symptoms were relieved by the use of depletory remedies; but as he was recovering from them, painful tumors began to make their appearance on the legs and arms. They became soft and fluctuating, and the integuments covering them assumed a livid hue. During the following week, an eruption of pustules took place on the forehead; the eyelids became swollen and painful, and an erysipelatous redness was diffused over the face. The alæ nasi became much tumefied, and an offensive mucus distilled from the nostrils. The patient recovered, but never perfectly, from these distressing symptoms. The general health continued cachectic to the period of his death, *eight months* from the commencement of his illness. On dissection the lungs were found to be most extensively tuberculated; the mucous lining of the nostrils and pharynx was inflamed and ulcerated, and insulated spots of inflammation were found scattered along the œsophagus, stomach and intestines. The trachea and its divisions were filled with a viscid mucus. In the muscles, as well as in the cellular substance of the extremities, there were numerous purulent tubercles. No purulent matter was found in any of the veins which were examined. The lymphatic vessels and glands in different parts were enlarged and apparently diseased.

The features of these cases will remind the experienced practitioner of the worst examples of ecthyma cachecticum.—*Rev.*

---

### HYDRATED PEROXIDE OF IRON, AN ANTIDOTE TO ARSENIC.

A German chemist, M. Bunzen, has lately announced that he has discovered an antidote to the virulent effects of arsenic, in one of the oxides of iron. His experiments have attracted much notice in France, and several of the leading toxicologists have already announced their assent to the practical conclusions which M. B. has drawn from his enquiries. M. Orfila is of opinion "that the importance of the discovery has not been exaggerated, and he therefore recommends a trial of the proposed remedy in cases of poisoning from arsenic." More recently MM. Miguel and Soubeiran have instituted an extensive series of examinations, the results of which we now propose to submit to our readers' attention.

To a middle-sized dog, 24 grains of white arsenic diffused through water were given. In ten minutes, the animal began to vomit, and the vomiting was soon followed with severe colicy pains, and with bloody alvine evacuations. After other ten minutes had elapsed it was made to swallow four drachms of the hydrated peroxide of iron diffused in water. During the rest of the day, the dog was feeble and exhausted; but next morning he was found to be quite lively, and in other two days he was as well as ever.

Eight grains of arsenic were given to a small dog. Ten minutes afterwards, when the effects of the poison had began to shew themselves, a dose of the peroxide was administered: "Des l'apres-midi ce chien paraissait tout-a-fait retabli."

These two experiments seemed to indicate that the peroxide acted as a direct antidote to the arsenical salt; but as in both, the animals were permitted at first to vomit freely, it may be objected that the poison was discharged from the stomach, before the peroxide was taken. To avoid therefore this objection, was the motive of the following experiments, in which the œsophagus of the animal was tied, after the arsenical dose had been swallowed. But first of all it was necessary to determine the average length of time, that a dog would live, after the mere operation of putting a ligature round the œsophagus. This was ascertained to be, from sixty to eighty hours. When a dose (twelve grains) of white arsenic were given before the œsophagus was tied, death followed in two hours, all the symptoms of poisoning (vomiting excepted) having been previously manifested.

Let us now ascertain what are the effects of exhibiting the hydrated peroxide, before the œsophagus is tied. Twelve grains of arsenic, diffused in water, were given to a small-sized dog, and immediately afterwards about two drachms of the peroxide of iron; the œsophagus was then tied, and the animal let free. For some time it made violent efforts to vomit; but, after the lapse of three hours, "les effets du poison avoient completement disparu." Twenty-four hours after the operation, the ligature was removed from the œsophagus, and the dog was made to swallow a little milk: no solid food could be swallowed; and, on the sixth day, it died.

To other two dogs, "d'assez forte taille," eighteen grains of the poison, and about three drachms of the peroxide were given, and the œsophagus was tied. One lived for seventy-eight hours, and the other for eighty-four hours after the operation. The results of these experiments are, that when the peroxide of iron was administered along with the poison, and the œsophagus then tied, the life of the animal was continued nearly as long as after the mere operation of tying the œsophagus, when no poison had been given; and in one case, in which the ligature was removed after the expiry of 24 hours, death did not ensue until the sixth day. It is impossible, therefore, to question the efficacy of the peroxide, as an antidote to the arsenious acid, at least when it was administered immediately after the poison had been swallowed.

A new set of experiments was now instituted, for the purpose of determining, whether the peroxide could in any measure counteract or diminish the virulent effects of the arsenic, after these had been once fairly established.

Eight grains of arsenic were given to a small-sized dog, and, two hours afterwards, a dose of the peroxide was administered. The animal died in a quarter of an hour after the peroxide had been injected into the stomach. To another dog, eighteen grains of the poison were given, and the œsophagus was then tied. Violent efforts at vomiting were induced. At the end of an hour, an opening was made into the œsophagus immediately beneath the ligature, and about three drachms of the hydrated peroxide, diffused in water, being injected, a second ligature was applied. This dog survived for ninety hours after the first operation. In other three experiments, conducted in the same manner, the animals lived for 24, 38, and 30 hours after the poison had been given them.

The counteracting effects, therefore, of the peroxide, even when a full hour had elapsed from the swallowing of the poison, are satisfactorily established by the results of these four trials. The prolongation of life, from two or three hours to between thirty and forty, attests the powerfully antidotal efficacy of the chalybeate. It was observed that, if the poison was blended with any fatty or oily matters, the peroxide of iron acted much less quickly and efficiently.

We shall now mention the results of some experiments made by M. Bunzen and other chemists, on the reciprocal chemical effects of the arsenic and peroxide out of the body.

If to a cold solution of one part of arsenic, twelve times as much of the hydrated peroxide (recently prepared) be added, the whole of the arsenic is immediately precipitated, in the form of an arsenite of iron. Such is the statement of M. Bunzen. That the neutralization may be complete, it is necessary that a much larger quantity of the peroxide, than exists in the precipitated arsenite, be present in the mixture. This is very apparent, if the experiment be performed in the following manner. To a solution of arsenic, let there be added only so much peroxide, as is known to be requisite to form a neutral arsenite. It will be found that a large portion of the arsenic remains behind in solution, and is not precipitated, however long the mixture be kept. But, if successive additions of the peroxide be made, more and more of the arsenic becomes precipitated, until, at length, no traces of the poisonous salt can be detected in the water. On the other hand, the precipitation is instantaneous and complete, if five times the quantity of the peroxide, that exists in the neutral arsenite to be formed, be added at once. Such are the effects of adding the peroxide of iron, to the arsenic in the state of aqueous solution. When, however, the arsenic is undissolved, the action of the peroxide, in forming a neutral arsenite of iron, is very slow and uncertain. The solution of the arsenic is quite indispensable to its rapid neutralization by the peroxide; and hence we can appreciate the injurious effects of any greasy matter in the stomach, in sheathing the arsenical salt from the action of the peroxide, and in therefore counteracting the antidotal efficacy of the latter.

The peroxide, which was used in the experiments of MM. Miguel and Soubeiran, was procured by the following process :—

The crystallized sulphate of iron was boiled in six times its weight of water in a platina or porcelain vessel: to the boiling solution, nitric acid was added gradually, as long as any ruddy fumes were disengaged. This was done for the purpose of bringing the oxide to the maximum of oxygenation. The liquor was diluted with water, and pure ammonia dropped in, to precipitate the peroxide, which is then to be washed by repeated affusions of distilled water, for the purpose of removing all traces of the ammoniacal salts. As the peroxide must be kept mixed with so much water, as to give the mixture the consistence "d'une bouillie claire," it is necessary to have some data, to determine the quantity which we use at any time. One part of the crystallized sulphate, treated as we have described above, yields exactly the third of its weight of hydrated peroxide, if this peroxide were in the dry state. The quantity, therefore, of sulphate which it is necessary to decompose, in order that we may have a sufficient dose of the peroxide, as an antidote to any given quantity of arsenic, is just thirty-six times the quantity of this poison. We have already mentioned how necessary it is that there should be a large excess of the peroxide, in order that the neutralization of the arsenic may be rapid and complete.

Let it be remembered that the peroxide should be fresh prepared, whenever we are about to use it as an antidotal remedy. If it has been dried and re-dissolved, its virtues cannot be depended upon.

As yet, no cases of poisoning with arsenic in the human subject, have been treated with the hydrated peroxide; but, from its unquestionable efficacy in the cases of the lower animals, we have most rational grounds for anticipating almost equal success in the human subject.—*Bulletin Gen. de Therapeutique.*

We have hesitated to publish the preceding memoir (which has lain in our desk for the last twelve months), as various writers had called in question the correctness of the statements therein made, and some had even alleged that the peroxide of iron was altogether without effect in counteracting the poisoning from arsenic. Within the last few months, however, we have discovered the following notices respecting this important subject.

In the Medicinishes Correspondenz-Blatt, Dr. Buzorini has narrated two cases of arsenical poisoning—not quite satisfactory in their details, we must confess—occurring in the human subject. Both patients, it is to be remembered, had vomited freely before the peroxide was administered. They recovered perfectly.

In the Journal de Medecine et de Chirurgie Pratiques, for last September, a case is reported by a M. Geoffroy. The details are lamentably imperfect. The hydrated tritoxide of iron was administered in about twenty minutes after the poison had been swallowed. The patient, a middle-aged man, vomited several times and he was also purged. No symptom of arsenical poisoning shewed itself; neither pain in the stomach, nor any sense of heat in the throat. He had been in a state of almost delirious excitement when he had swallowed the poison, and this delirium still continued. For the space of eight hours he was made to swallow at intervals tepid water in which the tritoxide had been diffused. On the following night he slept quietly, and next day all unpleasant symptoms had passed away.

It is supposed that this patient had swallowed not less than a drachm and a half of arsenic (!). Upwards of six ounces and a half of the ferruginous tritoxide had been administered. We (the Rev.) regret that we cannot agree with our French brother-critic that the preceding case " offre tout le degré d'authenticité qu'on peut desirer."

In the Journal des Connaissances Medico-Chirurgicales we find a still stronger testimony in favour of the antidotal powers of the tritoxide of iron. Four children, two of about seven years of age, and the other two of about five, had eaten some pieces of cake, which they had found lying on a mound near their father's house. The well-known symptoms of metallic poisoning soon developed themselves. M.M. Bineau and Majesté being summoned to their relief, readily detected in some pieces of the cake the presence of the arsenious acid, by means of some chemical tests, and by the peculiar garlic odour emitted when a portion of the cake was placed upon the live coals. The vomiting having been encouraged for some time, large doses of the tritoxide of iron were exhibited for several hours in succession. From three to five ounces in all were taken by each of the young patients. They all recovered.

On an accurate chemical examination of the cake (which, it was discovered, had been prepared for poisoning rats), the presence of the arsenious acid was unequivocally demonstrated. The urinal fluid tests were first employed, and then by sublimation in a small glass tube, the metallic arsenic was reproduced.

The method which M. Majesté recommends for preparing the tritoxide is by dissolving an ounce of iron-filings in a mixture of four ounces of nitric and four ounces of muriatic acid in a capacious vessel. To the solution, sixteen ounces of distilled water are to be added, and then two or three ounces of liquid ammonia are to be mixed with it. A hydrated tritoxide of iron is immediately precipitated, About twelve ounces of it are obtained by using the above-mentioned quantities of the different substances. It is of the consistence of a soft pap: one spoonful weighs about an ounce.

It deserves to be noticed that, in the cases of these children, the tritoxide was not administered for nearly three hours after the cake had been eaten, and that all the characteristic symptoms of arsenical poisoning had already manifested themselves.

The modus operandi of the tritoxide is by combining with the arsenious acid, and forming a compound which is insoluble, and therefore quite innocuous.

### Congenital Occlusion of the Vagina cured by a New Method.

The following very interesting case was recently communicated to the Institute by M. Amussat, one of the most active and able surgeons of the French Capital.

A girl fifteen years of age was brought from Germany to Paris for the purpose of consulting some of the leading surgeons there, MM. Boyer, Marjolin, Magendie and the narrator, for the relief of this distressing malformation. Her mother stated that she had enjoyed very good health until about two years ago, when she began to suffer severely from periodic attacks of pain in the abdomen and in the region of the kidneys. The first attack was on the 19th of June, 1831; it lasted for nine days. The second occurred five weeks afterwards and continued with occasional intervals of ease for nearly a month. During the following eight months, there were six attacks. At first they were attributed to an obstruction of the bowels; but it was soon discovered that this was not the cause, and on examination it was found that there was no trace of any vagina. The periodicity of the attacks was therefore attributed to the recurring efforts of Nature to bring about the catamenial discharge. When she was brought to Paris in February 1832, the following was the state of the patient. The abdomen was enormously distended, so as to equal almost the size in the sixth month of pregnancy: at the lower part was distinctly felt a firm, resisting, globular tumor, which was supposed to be the enlarged uterus. The external parts of generation appeared to be perfectly normal, but on separating the labia there was no appearance of any vaginal orifice; the meatus urinarius was situated considerably lower down than usual, occupying nearly the centre of the fossette of the vulva, and the concave surface corresponding to the orifice of the vagina was smooth and lined with a mucous membrane. Some difficulty was found in passing a catheter into the bladder, for it was requisite to direct it very obliquely upwards. On introducing the finger into the rectum, the catheter was felt very distinctly in consequence of the great thinness of the intermediate parts; and higher up the finger met with a large rounded tumor, which occupied the hollow of the pelvis: this tumor was tense, fluctuating, and seemed to have very thin parietes. The nature of the case could not be mistaken: the urethra, bladder and rectum were glued together; and the vagina, at least in two thirds of its length, "manquait absolument." The uterus in consequence had become enormously distended by the retained menstrual secretion. The prognosis was certainly very unfavorable. MM. Magendie and Marjolin were of opinion that the only thing to be done was to tap the uterus from the rectum, and thus relieve it from its contents. M. Boyer regarded the case as hopeless, founding his opinion on the result of all similar cases on record, and more especially in consequence of some recent cases known to him and to M. Marjolin, where an eminent surgeon, in attempting to separate the agglutinated parts with the knife, had perforated the bladder, and had caused the speedy death of the patients. He therefore strongly recommended that nothing should be done.

M. Amussat, while he agreed with the late venerable surgeon of La Charité in condemning any attempts with the knife, was unwilling to abandon the case as irremediable. Two cases of imperforation of the vulva had recently occurred in his practice, where he had succeeded in re-establishing the vaginal passage by gradually tearing asunder the unnatural union between the urethra and the rectum with blunt instruments and with the fingers, and he thought that the same method was worthy of trial in the present instance. Having obtained the consent of the girl and her mother, he proceeded on the 29th of February to put it into practice. He used at first a large sized straight sound, and applying its rounded end to the fossette of the vulva immediately below the opening of the meatus urinarius, he pressed it with considerable force in the direction of the vagina,

" comme pour faire un trou." He repeated this operation with the point of his finger, keeping all the while the fossette stretched by drawing the perineum strongly backwards, and the meatus urinarius upwards. Having continued the *boring* for some time he found that " il resta un trou, ou plutôt une impression profonde sans dechirure, ni effusion de sang." A small piece of prepared sponge was placed in the hollow (enfoncement) thus made, and it was retained in its place by a bandage. Three days subsequently, the operation was repeated. M. Amussat used two fingers this time in making the pressure in the line of the vagina, and he employed such force as to occasion a slight laceration of the parts, and consequently some effusion of blood. " Ce decollement fut tres douloureux, mais profitable."

The dilatation thus obtained was prevented from contracting by the insertion of a piece of sponge. For the following three days, the same manœuvre was repeated, and with more and more effect, the two fingers being always introduced with the points of their nails close to each other and then gradually separated, so as to tear gently asunder the agglutinated canal. By having the catheter in the urethra and a finger in the rectum, the risk of wounding these parts was quite obviated. Already an inch and a half or nearly two inches of the canal had been gained, and the finger could then distinctly feel the distended uterus. For four days the patient was allowed to remain quiet without any thing more being done, and on the 9th of March M. Amussat perforated the distended tumor, with a small trocar using his finger as a director. On withdrawing the stylet, only a few filaments of dark-coloured blood escaped through the canula. He had recourse therefore to a bistoury, which he conveyed along his finger, and which he cautiously plunged into the tumor, the walls of which were found to be extremely firm and resisting. Some dark, glutinous and thick blood, more like thick chocolate than any thing else, flowed out. The aperture made with the bistoury being small, he attempted to enlarge it with the point of the finger, but this was impracticable in consequence of the hardness of the tissues. He therefore again introduced the bistoury and enlarged the incision. Immediately a stream of glutinous blood escaped. The finger could now be passed into the cavity of the tumor, which was found to have a smooth surface ; but the pain occasioned by this proceeding was so severe, that the patient could scarcely endure it. The finger being withdrawn, M. Amussat divided with the knife a few bridles which prevented the free dilatation of the incision, and immediately ten or twelve ounces of dark blood were discharged. The size of the abdomen was very sensibly diminished. An elastic gum canula was left in the orifice and made secure in its place by a bandage passed round the pelvis. The constitution suffered a good deal during the next twenty-four hours. An injection (we suppose of tepid water) had been twice practised into the cavity of the tumor, and had brought away a little glairy blood ; nearly two pints in all had escaped since the operation. On the following two days the patient was extremely low and oppressed, and feverish symptoms, attended with vomiting, colicy pains and bilious diarrhœa came on. There was great prostration of all the vital powers ; the pulse was rapid and feeble ; there was an acute fixed pain in the left iliac fossa ; the whole abdomen was tender and inflated, and there was a great tendency to stupor. M. Magendie being summoned in consultation, expressed great alarm for the result of the case. The employment, however, of leeches to the left groin, and of mercurial ointment rubbed over the whole abdomen, gave very decided relief. The patient, suffering much from tympanitic distention of the bowels, a long elastic canula was passed into the rectum high up, and gave exit to a large quantity of gas. On the 13th, the patient was somewhat easier, and the edges of the incised uterus were found to be suppurating freely. As the pain in the left groin returned towards evening, fifteen leeches were again applied; but the patient was so weak, that it was necessary to stop the bleeding from the bites. During the following week, the state of the patient did not improve much; there was always a distressing feverishness, and extreme languor and depression. A large canula was left occasionally for twelve or twenty-four hours in the vagina. On the 18th, there was a bilious diarrhœa, and with the motions a quantity of blood, altogether similar in appearance to what had escaped from the vagina after the operation, was discharged per anum. This phenomenon was repeated several times for some days in succession. From this date, however, to the 1st of April, the general condition of the patient very sensibly mended, although there was still great weakness, and tendency to attacks of evening feverishness. By the aid of canulæ, gradually used larger and larger, the artificial vagina had become considerably wider. The swelling and tenderness in the left groin was much less, and the uterus had resumed its normal dimensions. Although there were occasional relapses of pain and of general distress, the patient was so well by the 23d of April, that she was able to leave Paris for her native place, on the banks of the Rhine.

By a letter received from the mother, M. Amussat learned that, a few days after reaching home, her daughter menstruated. The discharge was pale, and continued for nearly twenty-four hours. Ten days afterwards the catamenia reappeared, and lasted only for six hours. From this period they did not return till the month of July, in the first week of which, after suffering greatly from acute pain in the back and through the pelvis, the patient again menstruated. The quantity of the menses was profuse, and they were mixed with coagula, like pieces of softened liver. During the following four months, the patient suffered greatly at each catamenial period, and in October she was taken so seriously ill, that her mother was alarmed that she might have an attack similar to what occurred a few days after the operation, when the discharge of blood per anum took place. It was not until the February of 1833, that the girl's health was fairly re-established. From this date, the catamenia returned regularly every four weeks, without being attended with pain or any inconvenience, and the patient, from being weak and emaciated, recovered her strength and embonpoint.

M. Amussat remarks that, in the successful treatment of the preceding case, he was marvellously assisted by Nature having induced an adhesion between the parietes of the distended uterus and the large intestine, and having thus prevented the effusion of its contents into the abdominal cavity—an accident which otherwise must, in all probability, have taken place at the time when the copious discharge of blood per anum occurred.

[We are by no means satisfied by the details of the report, that the discharge of blood from the bowels, on the 18th of March, was the result of any rupture of the uterus or of the Fallopian tube into the large intestine, as alleged by M. Amussat. The discharge may have been quite independent of such an accident.—Rev.*)

In July, 1834, M. Amussat's patient re-visited Paris. She was in perfect health; all the functions, and especially that of menstruation, being performed "à merveille." Madame Boivin, being commissioned to make an examination of the parts, drew up the following report:—

"The vagina is nearly an inch and a half in depth; its surface is smooth, and altogether similar to the inner surface of the labia. There is no appearance of labia minora or nymphæ. The

* The reason given by M. Amussat for his opinion, viz. the circumstance of the blood discharged per anum being quite similar to what had escaped per vaginam after the operation—is to us unsatisfactory. We are well aware that extra-uterine conceptions, and abscesses of the Fallopian tubes and of the ovaria, have been discharged by the bowels; but, without further proof, we cannot readily admit this explanation in the present case.

clitoris is well formed. At the extremity of the vagina, the finger discovers a small cicatrix, which feels like the umbilicus of a new-born infant; but it is not provided with any aperture. Upwards and behind the symphysis pubis, is felt an oblong elastic substance, about half an inch in diameter: this I took at first to be the cervix uteri; but, on a more minute examination, it proved to be formed by the anterior wall of the vagina, pushed down and somewhat displaced by the dilated urethra.* At the extremity of the vagina, where the small cicatrix is felt, I find with the speculum an opening, through which I can pass a large probe for five or six lines. The finger introduced into the rectum discovers a small oblong body, terminating upwards in another one of larger dimensions, which is no doubt the uterus. This organ seems to me to be perfectly normal."

In a subsequent consultation between MM. Amussat, Marjolin, and Magendie, they all agreed—" qu'il existait un petit vagin, qui s'etait formé aux depens des petites et meme des grandes levres, la muqueuse ayant eté attirée dans le vagin artificiel par la cicatrice; et qu'au fond de ce vagin on decouvrait un trou, ou fistule, qui conduisait à la matrice, et par lequel l'ecoulement des regles se faisait librement." MM. Marjolin and Magendie cheerfully expressed their decided conviction, that the life of the patient had been saved by the operation which M. Amussat had performed; an operation, the result of which was the more creditable to his skill, as it had never, to their knowledge, succeeded before; but it was very doubtful, they added, whether the unfortunate girl was, or ever could be in a marriageable condition.

There was nothing, indeed, to prevent the occurrence of conception; but the imperfect state of the vagina must expose the patient to extreme danger at the period of delivery, if gestation should advance to the full term. The fistulous opening in the uterus and the utero-vaginal canal (le trajet de la fistule utero-vaginale), can perhaps never dilate sufficiently to permit a full-grown fœtus to pass.

The use of bougies, to be gradually increased in size, was recommended with the view of increasing the size of the utero-vaginal canal. On the 29th Nov. 1835, M. Amussat received from the patient's mother a letter, wherein she states—" My daughter keeps her health well; she is very regular, and suffers no pain at the periods. Bougies of about half an inch in thickness can be introduced to the depth of four inches and a half. The fistulous cicatrix is now easily dilatable."

Such are the particulars of this very interesting case at the period when the report of it was submitted to the French Institute.

M. Amussat strongly advises surgeons to attempt the cure of all such cases in the manner which he practised in the preceding one;—by cautious laceration and dilatation of the united parts, rather than by division with a cutting instrument. It will have been observed that two of the surgeons who attended this case, recommended that the uterus should be punctured from the rectum, for the purpose of giving exit to the retained catamenia. M. Amussat attained the same object in a much more scientific and satisfactory method; and his example deserves to be followed in similar cases. M. Boyer, in the tenth volume of his Surgery, has treated, at considerable length, of the various congenital defects of the female organs of generation, and has alluded to the practice to be followed in occlusion of the vagina. He says: " death is the inevitable consequence of the accumulation of the menses in the uterus, when there is a complete absence of the vagina. The only method of preventing the fatal event is to puncture the uterus, either from the perineum (provided there be a sufficient thickness of the septum between the rectum and the

* The patient, it appears, had committed the awkward mistake of having introduced pieces of sponge into the meatus urinarius instead of into the vagina, and had thus caused a very considerable dilatation of the urethra for about an inch of its extent.

urethra) or from the rectum. The operation is a very hazardous one: the bladder has been perforated in more than one case, and the patient has necessarily perished in consequence; and when this accident has been avoided, I have known the patients die from inflammation of the uterus and of the adjoining parts." M. Amussat mentions that, in the unpublished Archives of the Royal Academy of Medicine, there is a history of a case of occlusion of the vagina, successfully treated by Dr. Villiaume of Metz, who divided the vulva with a scalpel to the depth of an inch, and then perforated the distended uterus with a bistoury. In this case, there appears to have been a considerable thickness of substance in the septum, between the rectum and the urethra.—*Gazette Med. de Paris.*

---

SINGULAR CASE OF CONGENITALLY IMPERFORATE VAGINA; MENSTRUATION BY THE URETHRA: SUCCESSFUL OPERATION.

Dr. Coste, the "premier chirurgien-chef interne" of the Hôtel Dieu at Marseilles, is the narrator of the case, the abridged report of which we now communicate. A young woman, 21 years of age, exhibited the following strange state of the generative organs. The clitoris was greatly enlarged, so as to resemble the penis of a boy at 12 or 14 years old: the situation of the meatus urinarius was normal, but through it the menses, as well as the urine, were regularly voided. The girl had menstruated regularly since she was 13 years of age. There was no appearance of a vagina; its place being occupied with an integument covered with hair, and exhibiting a raphé along the centre. The labia pudendi were merely rudimentary. The author adds that in the site of the right inguinal aperture there was "un testicule tres facile à reconnaitre par sa forme, sa dureté, et la douleur qu'y determinant la pression." (There might have been a *something*, but surely it was not a *testicle.—Rev.*)

The features and general characters of the body and limbs were those of a female, and her "gouts et penchans" were in conformity. The young lady "aimait tendrement" a young gentleman; and it is not wonderful that she was "pleine d'espoir et de joie," at my proposal to fit her for matrimonial duties. Dr. Coste, having first introduced a canula along the meatus urinarius, ascertained that it might be passed in two directions; in one towards the bladder, and in the other, towards the uterus. It was therefore deemed almost quite certain that the vagina opened into the urethra, at a short distance from the opening of the meatus. The existence of the catamenia afforded a sufficient proof that a uterus was present. On cutting to the depth of an inch in the line of the raphé he could not discover any trace of the vagina; he therefore determined to slit the under wall of the urethra from its orifice to the point, where the canula seemed to glide from the urethra in a direction towards the uterus. The finger being now introduced to the bottom of the wound, he discovered in the middle of a mucous cul-de-sac a rounded projecting object, which proved to be the os tincæ. The enlarged clitoris was then amputated by one stroke of the scalpel. A large roll of linen well smeared with oil was left in the newly-formed vagina, and the patient was put upon a rigorous antiphlogistic regimen. The roll of linen was withdrawn and replaced daily. At the end of two months the cicatrization of the divided urethra was complete; but the use of gradually enlarged bougies was continued for some time afterwards, in order to prevent the tendency of the artificial vagina to contract. The catamenia returned with their accustomed regularity, and were now discharged per viam naturalem. This damoiselle was married, we are told, eight months after the operation was performed: "la jeune epouse m'a avouée que le coit s'executait sans la moindre difficulté, et que les sensations qu'elle eprouvait etaient vives." (A delicate confession!)—*Journ. des Connoiss. Medicales.*

### Human Embryo vomited by a Child.

Dr. Vourons in a letter recently read to the Royal Academy narrates this extraordinary occurrence. " I have recently had occasion to observe in the town of Syra, where I reside, a most curious phenomena. A boy, three years of age, who had suffered for a considerable time from the symptoms of vermination, vomited after repeated efforts a human embryo. I have ascertained by careful examination that it was truly an embryo; and the police, having suspected some fraud, has instituted a minute enquiry, the result of which is quite conclusive in favour of the facts as now stated." When this letter was read to the Academy, M. Geoffroy St. Hilaire (whose authority on such subjects is unquestionably very high), stated that he had lately had an opportunity of witnessing a similar example. He was on the spot at the time that the strange substance was vomited; and since his return to Paris he has examined it along with M. Milne Edwards, and has determined that it was a " Mole," without any distinction of members or organs.

---

### On the more Common Causes of Death in New-Born Infants.

The following may be considered as the most frequent of these causes. When, after labour has once fairly commenced, the pains either cease entirely, or they abate and intermit so much, that the delivery is rendered very tedious and protracted—when convulsions occur during the progress of labour—when the navel-string is either too short, or has become twisted round the neck, or some other part of the child—when the navel-string is prolapsed along with the head and is compressed between it and the walls of the pelvis, so that the circulation is interrupted—when the placenta becomes partially or entirely detached before the expulsion of the child, and hæmorrhage is thus induced—when the os uteri, or the constrictor muscle of the vagina is spasmodically contracted (as is apt to occur in first labours) round the neck of the child—or lastly, when the pelvis is so small that the exit of the head is delayed for a great length of time. Such are the most common causes of the death of the child during the act of labour. It may be useful now to allude briefly to those from which the child may perish immediately after the delivery has been accomplished :—when the child is exceedingly feeble and exhausted—when the child is born closely invested with the membranes, and the head is not disengaged from these sufficiently soon —when the child is at birth apparently still-born, and the appropriate means of resuscitation are not adopted early enough—when respiration is interrupted by an inordinately-sized thymus gland pressing on the trachea, or by any defective state of the pulmonary apparatus—when the mouth is plugged up with viscid mucus, or, as sometimes happens, the tongue falls backwards and closes up the fauces—when the child is seized with apoplexy—when the mother is in a state of syncope at the moment of delivery—or when, from the ignorance, or the wilful neglect of the attendants, the necessary means are not adopted for the preservation of the child.—*Wildberg's Jahrbuch der gesammten Staatsarzneikunde.*

---

### Treatment of Ossifications occurring in Muscular Texture.

The ossifications to which the author alludes are what the German surgeons call " exercise—or drill bones," from their occurring in the deltoid muscles of the arms in young recruits. They are caused by the pressure of the musket on the shoulder. Richter published, a few years ago, a pamphlet on this subject, and described very accurately the progress of the change, from a phlegmonous hardening of the skin and subcutaneous cellular tissue, to a gradual bony induration of the more superficial layers of the deltoid muscle. The following is a case which we find reported by one of the Prussian army surgeons.

In the left shoulder, over the anterior side of the joint, of the fusileer K. of the 31st infantry regiment, an "exercise-bone" had formed. It was about two inches and a half long, and nearly an inch broad; and it felt to be as hard as a piece of stone. It had gradually formed during the two or three months drilling, which the young soldier has to pass through, when he enters the army, and had been caused no doubt by the irritation induced by the pressure of the musket on the point of the shoulder. The surgeon of the regiment had at first prescribed several applications of the cupping-glasses, and the infrication of mercurial ointment on the swelling, night and morning. After a fortnight's continuance of these means, half a tea-spoonful of the tincture of iodine was ordered to be rubbed upon the joint twice a day; and to increase the activity of the absorbents, the cold douche bath was used, in conjunction with active friction. By a steady perseverance in the employment of these remedies, the hard swelling was gradually dissipated, so that the young man could shoulder his musket, without suffering the slightest inconvenience.

In some cases, the mere discontinuance of the drill for several months is followed by a dispersion of the indurated swelling. It appears that the excision of the bony mass has been occasionally resorted to; but we should think that this severe remedy is seldom advisable. Might not the evil be prevented by the recruit bathing the shoulder with some spirituous discutient lotion during the first months of his drilling?—*Medizinische Zeitung*.

